# Novel Gut-Based Pharmacology of Metformin in Patients with Type 2 Diabetes Mellitus

**DOI:** 10.1371/journal.pone.0100778

**Published:** 2014-07-02

**Authors:** Antonella Napolitano, Sam Miller, Andrew W. Nicholls, David Baker, Stephanie Van Horn, Elizabeth Thomas, Deepak Rajpal, Aaron Spivak, James R. Brown, Derek J. Nunez

**Affiliations:** 1 Immuno-Inflammation Unit, GSK R&D, Stevenage, Herts, United Kingdom; 2 Quantitative Sciences, GSK R&D, Stevenage, Herts, United Kingdom; 3 Safety Assessment, GSK R&D, Ware, Herts, United Kingdom; 4 Target and Pathways Validation, GSK R&D, Upper Providence, Pennsylvania, United States of America; 5 Computational Biology, GSK R&D, Upper Providence, Pennsylvania, United States of America; 6 Enteroendocrine Discovery Unit, GlaxoSmithKline R&D, GSK R&D, Research Triangle Park, North Carolina, United States of America; Daping Hospital, Third Military Medical University, China

## Abstract

Metformin, a biguanide derivate, has pleiotropic effects beyond glucose reduction, including improvement of lipid profiles and lowering microvascular and macrovascular complications associated with type 2 diabetes mellitus (T2DM). These effects have been ascribed to adenosine monophosphate-activated protein kinase (AMPK) activation in the liver and skeletal muscle. However, metformin effects are not attenuated when AMPK is knocked out and intravenous metformin is less effective than oral medication, raising the possibility of important gut pharmacology. We hypothesized that the pharmacology of metformin includes alteration of bile acid recirculation and gut microbiota resulting in enhanced enteroendocrine hormone secretion. In this study we evaluated T2DM subjects on and off metformin monotherapy to characterize the gut-based mechanisms of metformin. Subjects were studied at 4 time points: (i) at baseline on metformin, (ii) 7 days after stopping metformin, (iii) when fasting blood glucose (FBG) had risen by 25% after stopping metformin, and (iv) when FBG returned to baseline levels after restarting the metformin. At these timepoints we profiled glucose, insulin, gut hormones (glucagon-like peptide-1 (GLP-1), peptide tyrosine-tyrosine (PYY) and glucose-dependent insulinotropic peptide (GIP) and bile acids in blood, as well as duodenal and faecal bile acids and gut microbiota. We found that metformin withdrawal was associated with a reduction of active and total GLP-1 and elevation of serum bile acids, especially cholic acid and its conjugates. These effects reversed when metformin was restarted. Effects on circulating PYY were more modest, while GIP changes were negligible. Microbiota abundance of the phylum Firmicutes was positively correlated with changes in cholic acid and conjugates, while Bacteroidetes abundance was negatively correlated. Firmicutes and Bacteroidetes representation were also correlated with levels of serum PYY. Our study suggests that metformin has complex effects due to gut-based pharmacology which might provide insights into novel therapeutic approaches to treat T2DM and associated metabolic diseases.

**Trial Registration:**

www.ClinicalTrials.gov NCT01357876

## Introduction

Metformin, a biguanide derivate, is the first line of treatment in patients with type 2 diabetes mellitus (T2DM), in conjunction with lifestyle modification, as indicated in the guidelines issued by the American Diabetes Association and European Association for the Study of Diabetes [1]. Metformin enters hepatocytes through the organic cation transporter-1 (OCT-1) transporter, and there it is thought to alter mitochondrial function and AMP kinase (AMPK) activity [2], resulting in decreased hepatic glucose production and glucose lowering, while AMPK activation in skeletal muscle may increase glucose utilization [3]. In addition, metformin improves the lipid profile [4], restores ovarian function in polycystic ovary syndrome [5], reduces fatty infiltration of the liver [6], and lowers microvascular and macrovascular complications associated with T2DM. Recently, metformin has been proposed as an adjuvant treatment for cancer [7], as a treatment for gestational diabetes and for the prevention of T2DM in pre-diabetic individuals [8].

Mitochondrial function and AMPK activity in liver and skeletal muscle have received much attention as potential mechanisms by which metformin has its beneficial effects. In contrast to oral dosing, intravenously-administered metformin does not improve glucose metabolism [9], suggesting that other organs, such as the gastrointestinal tract, may be the principal site of action of this drug, although those mechanisms are unclear at present. Glucagon-like peptide-1 (GLP-1) and glucose-dependent insulinotropic peptide (GIP), secreted by enteroendocrine cells in the gut, are important determinants of glucose disposal following a meal [10]. In T2DM, fasting and post-prandial circulating levels of GIP are normal or increased, but the β-cell response to this peptide is diminished. In contrast, β-cells remain responsive to the insulinotropic action of GLP-1, but meal-stimulated GLP-1 increases are diminished [11]. Enteroendocrine cells also secrete peptide tyrosine-tyrosine (PYY), a peptide implicated in the control of food intake [12]. Dipeptidyl peptidase-IV (DPP-IV) is the protease responsible for the rapid degradation of active GLP-1_7–36_ and GIP_1–42_, and for the conversion of PYY_1–36_ to PYY_3–36_ [14], [13]. Some have reported that metformin increases circulating active GLP-1_7–36_ [14], [15], [16] or total GLP-1 [17], [18], while others describe a lack of effect on DPP-IV [19] or variable inhibition. Metformin may also facilitate the secretion of active GLP-1_7–36_, perhaps through a muscarinic receptor subtype 3/gastrin-releasing peptide pathway [20]. There is also evidence that metformin may reduce bile acid reabsorption in the distal ileum [21], and this may result in greater availability of bile acids in the colon for interaction with the farnesoid-X receptor (FXR) [22] and TGR5 receptors [23].

Increasing evidence links changes in the gut microbial community or the microbiome to disease severity of obesity and T2DM [24]. Moreover, there is growing appreciation of the effects of drugs, besides antibiotics, on gut microbial communities [25]. Although metformin is one of the most widely prescribe drugs for the treatment of T2DM, there is little information on its effects on the human gut microbiome. Intriguingly, a recent study found that metformin does alter the gut microbiota in the worm *Caenorhabditis elegans* [26]. A recent preliminary case report found that a cobiotic comprised of inulin, blueberry extract and beta-glucan can provide relief from the diarrhoea side-effects associated with metformin [27], further suggesting potential interactions between metformin and the human gut microbiome.

In this study we aimed to characterize in more detail the gut-based pharmacology of metformin in patients with T2DM by relating glycemic control to bile acid excretion and microbiota changes. Because of the complex pharmacokinetics of metformin in the gut [28], we employed the paradigm of withdrawing and restarting metformin, following the rise and fall of fasting capillary blood glucose (CBG) as a marker of metformin effect. With this approach we observed that metformin alters bile acid metabolism, gut peptide secretion and the gut microbiome, actions that may underpin some of the pleotropic benefits of this important anti-diabetic medicine.

## Methods

The protocol for this trial and supporting TREND checklist are available as supporting information; see Checklist S1 and Protocol S1.

### Ethics Statement

This exploratory, unblinded study (www.clinicaltrials.gov, NCT01357876) was performed in accordance with ICH Good Clinical Practice Guidelines, the principles of the 2000 version of the Declaration of Helsinki [29] and all subject privacy requirements. The study protocol was approved by the protocol review panel at GlaxoSmithKline and the Cambridgeshire Local Research Ethics Committee (10/H0306/45), and all subjects gave written informed consent before enrolment in the study. The study was conducted in the GlaxoSmithKline Clinical Unit, Addenbrooke's Hospital, Cambridge, UK.

### Study Design

A total of fourteen male (n = 2) and female (n = 12) patients with T2DM were recruited by direct advertisement. Inclusion criteria at screening were: age 18–70 years of age and glycosylated haemoglobin level (HBA1c) between 6.5% and 8.5%. All subjects had to be on stable dose of metformin of ≥1000 mg/day for more than 3 months. Two patients (both males) withdrew consent for personal reasons before completion of the study procedures. As full data sets were not available for these two subjects, they were replaced by 2 new (female) subjects to provide complete data for 12 subjects.

We followed the rise and fall of fasting CBG using a home monitoring glucometer (ACCU-CHEK *Aviva* glucometer, Roche Diagnostics) during metformin washout and re-introduction to establish the timing of the study visits. The washout period refers to the period from the end of Visit 1 (time when metformin treatment was stopped) to the end of Visit 3 when metformin treatment was resumed. While the time between the Visit 1 s and 2 was set at seven days, the time between Visits 2 and 3 was determined by the individual subject's rate of change of CBG (to reach the targeted increase of 25% from the average CBG measurements prior to Visit 1), and thus it varied depending on the individual differences in the loss of the glycemic effect of metformin.

**Figure 1:**
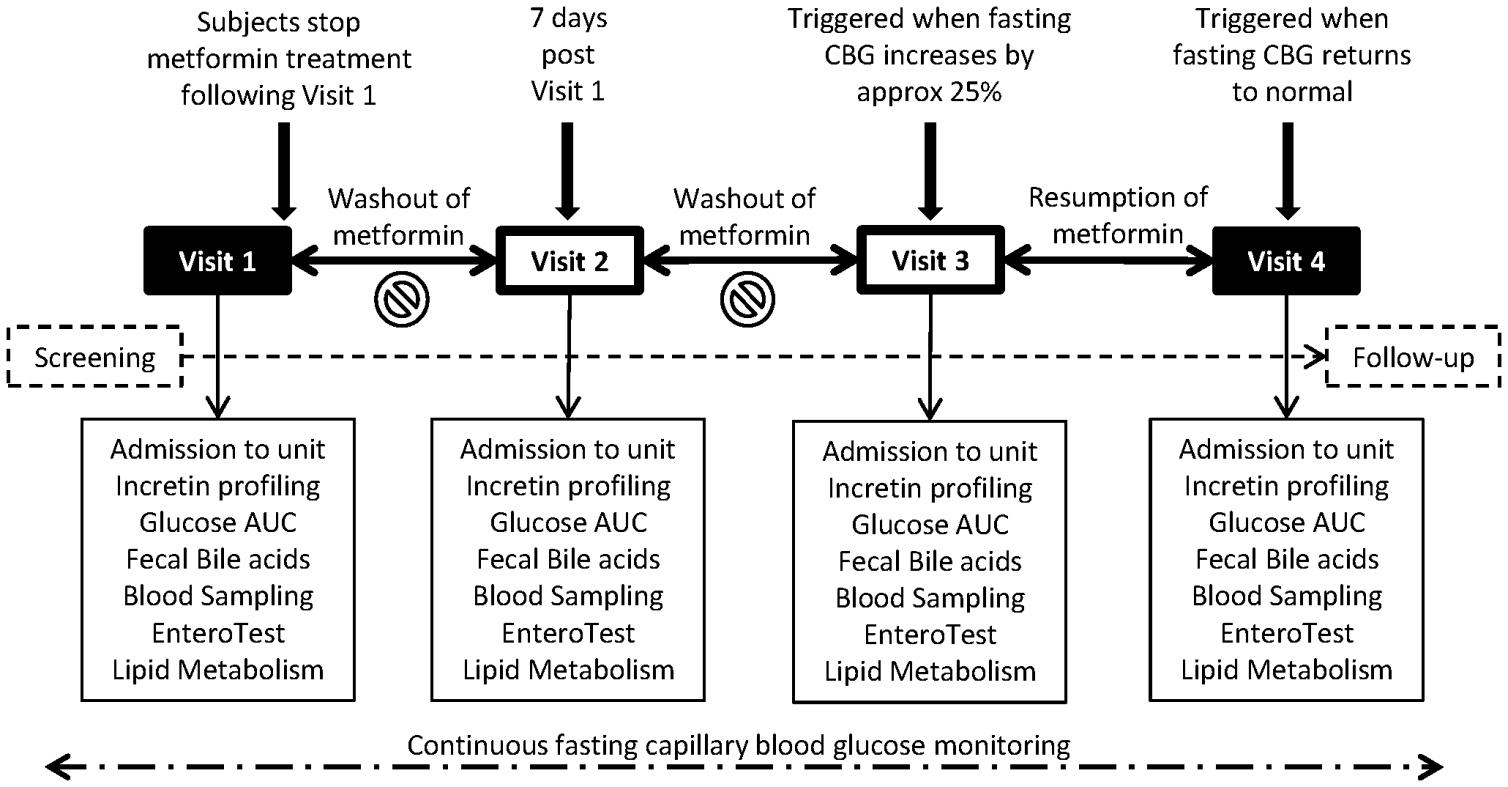
Schematic of the study design. Subjects were studied at 4 time points: (i) at baseline on metformin, (ii) 7 days after stopping metformin, (iii) when fasting blood glucose (FBG) had risen by 25% after stopping metformin, and (iv) when FBG returned to baseline levels after restarting the metformin.

Subjects remained at home between the visits taking their usual dose of metformin, and were studied on four separate occasions as follows (Figure 1):

- Visit 1: Whilst on their usual stable dose of metformin (baseline state);
- Visit 2: Seven days after stopping metformin treatment;
- Visit 3: When fasting CBG had increased by 25% from the average baseline pre-Visit 1 metformin washout level, but no more than three weeks from the start of the washout period;
- Visit 4: At the time when fasting CBG had returned to the average baseline pre-Visit 1 washout level after the same dose of metformin had been re-introduced.

At each scheduled visit, the subjects were admitted to the clinical unit the evening before the study visit. After an overnight fast, they underwent the following procedures on the day of investigation:

- Repeated post-prandial blood sampling at 0 (fasted) and 30, 60, 90, 120, 150, 180, 240, 270.300. 360, 600, 630, 660 and 720 min for the measurement of serum glucose, insulin, bile acid profiles and enteroendocrine peptides (total GLP-1, active GLP-1_7-36_, total PYY and GIP);
- Faecal sampling for bile acids and gut microbiome analysis;
- Sampling of upper small intestinal bile using the Entero-Test string. The subjects swallowed a capsule with the string at 22:00 the evening before, and the string was retrieved prior to breakfast, using the procedure described by Guiney *et al*. [30];
- Fasting plasma samples for metformin concentrations.

Standardized meals were provided on the study days: breakfast at approximately 08:00 (0 h), lunch at approximately 12:00 (4 h) and dinner at approximately 18:00 (10 h). Metformin was dosed at approximately 07:55 on all study days.

### Methodology

Pharmacodynamic and pharmacokinetic venous blood samples were collected in serum separator and EDTA tubes. Plasma samples were rapidly separated by centrifugation at 4°C. The serum separator tubes were left on the bench at room temperature for the blood to clot before centrifugation. Plasma and serum was stored at −70°C until they were analyzed. Faecal samples were collected while in the unit and stored at −70°C until they were analyzed.

Plasma total and active GLP-1_7–36_, total PYY and GIP concentrations and plasma glucose levels were measured by BioAgilytix Labs (Durham, NC) using the assays shown in Table 1.

**Table 1:** Glucose and peptide assays.

Assay	Manufacturer	Catalog Number	Notes on the Assay
**Active GLP-1**	Meso Scale Discovery	K151HZC	Electrochemiluminescent multiplex immunoassay. Standard curve range is 3.13 – 200 pg/mL
**Total GLP-1**	Meso Scale Discovery	Multiplex assay with glucagon and insulin, catalog number K15160C	Electrochemiluminescent multiplex immunoassay. Standard curve range is 3.13 – 200 pg/mL
**PYY**	Millipore	EZHPYYT66K	Sandwich enzyme-linked immunosorbent assay. Standard curve range is 40 – 2000 pg/mL
**GIP**	Millipore	EZHGIP-54K	Sandwich enzyme-linked immunosorbent assay. Standard curve range is 7.18 – 1000 pg/mL
**Glucose (2300 STAT Plus Glucose Analyzer)**	YSI	Glucose/lactate standard, catalog number 2747	Automated instrument for measuring glucose concentration in plasma or serum

Venous serum samples, Entero-Test bile samples and faecal samples were analyzed for primary, secondary and conjugated bile acids. Bile acid (BA) standards, cholic acid (CA), chenodeoxycholic acid (CDCA), deoxycholic acid (DCA), glycochenodeoxycholic acid (GCDCA), glycocholic acid (GCA), glycodeoxycholic acid (GDCA), lithocholic acid (LCA), taurochenodeoxycholic acid (TCDCA), taurocholic acid (TCA), taurodeoxycholic acid (TDCA), taurolithocolic acid (TLCA), tauroursodeoxycholic acid (TUDCA) and ursodeoxycholic acid (UDCA), were purchased from Sigma Aldrich (Gillingham, UK) and were ≥95% purity. The internal standards, cholic acid (24-^13^C) and deoxycholic acid (24-^13^C), were obtained from Cambridge Isotope Laboratories (Massachusetts, USA) and were 99% pure.

#### Preparation of Stock, Calibration Standard and Internal Standard Solutions

Bile acid stock solutions were prepared by dissolving each individual bile acid in the appropriate volume of methanol to obtain a concentration of 10 mg/mL. Thirteen-point calibration curves ranging from 1 ng/mL to 20 µg/mL were prepared by diluting appropriate volumes of each of the bile acid standard stock solutions with LC-MS chromatographic solvent (75:25 methanol:H_2_O containing 0.01% formic acid and 5 mM ammonium acetate).

#### Serum Sample Preparation

150 µL of neat human serum sample was dispensed into 450 µL of internal standard extraction solution in a 96-deep well plate (Agilent Technologies, Cheshire, UK) and mixed thoroughly for 10 minutes on a Stuart SSM1 mini orbital shaker (Bibby Sterlin Ltd, Staffordshire, UK). The samples were then centrifuged at 3220rcf for 10 minutes to pellet the precipitated protein content. Aqueous calibration standards, the solvent double blank and the solvent blank were prepared in the same manner. 400 µL of supernatant was removed from each sample, calibration standard and blank and transferred into a 96-deep well plate. The supernatant samples were evaporated to dryness using a Genevac HT-4 series II sample evaporator (Genevac, Suffolk, UK). The samples were reconstituted in 130 µL of LC-MS chromatographic solvent (75:25 methanol:H_2_O containing 0.01% formic acid and 5 mM ammonium acetate), sealed and mixed for 10 minutes on a mini orbital shaker to dissolve the extract within the wells of the plate prior to analysis.

#### Faecal Sample Preparation

After thawing, approximately 100–200 mg of faecal sample was weighed into individual Chromacol 5-SV 5.0 mL glass round bottom vials (Chromacol, Hertfordshire, UK) and the weights were recorded. One mL of LC-MS chromatographic solvent (75:25 methanol:H_2_O containing 0.01% formic acid and 5 mM ammonium acetate) was dispensed into each sample and the samples were mixed by vortexing and then centrifuged at 3220rcf for 15 min to recover faecal sample adhering to the vial wall. Each sample was then homogenised for approximately 20 sec using an Ultra Turrax T-25 homogeniser (Fisher Scientific, Loughborough, UK). A second 1.0 mL aliquot of chromatographic solvent was dispensed into the samples and the samples were homogenized for a further 20 sec. The samples were centrifuged at 3220rcf for 5 minutes. The clear faecal supernatant samples (1.5 mL) were removed and collected into glass scintillation vials. These steps were repeated and the supernatant samples were combined yielding a total volume of 3.0 mL. 30 µL of sample was then dispensed into 270 µL of LC-MS chromatographic solvent to produce a diluted sample for analysis. 15 µL of Internal Standard spiking solution containing ^13^C cholic acid and ^13^C deoxycholic acid at 100 µg/mL was dispensed into 300 µL of diluted sample, the calibration standards and the blanks in screw cap LC vials (Agilent, BÖblingen, Germany) and mixed by vortexing prior to analysis.

#### Entero-Test Bile String Sample Preparation

Immediately after removal each Entero-Test string was stored in a 50 mL falcon tube (Becton Dickinson, New jersey, USA) and frozen at −70°C until they were processed.

Each Entero-Test string was placed into the barrel of a BD Plastipak 5 mL hypodermic sterile syringe (Becton Dickinson, Madrid, Spain) and the bile was recovered by re-inserting the plunger and expelling the sample into a 20 mL glass collection vial (Wheaton, New Jersey, USA). 2.5 mL of HPLC chromatographic solvent was drawn into the syringe and the string was washed by manual agitation. The string washing was dispensed into the glass vial. This step was repeated a total of three times. Four mL of solvent was used to rinse the original 50 mL falcon tube (Becton Dickinson, New jersey, USA) in which the string was stored and the washes were combined. 8.5 mL of buffer was added to the glass vial to give a final volume of 20 mL of chromatographic solvent containing the chemicals extracted from the sting. Serial dilutions of this were performed by dispensing 50 µL of sample into 450 µL of chromatographic solvent and 20 µL of this sample into 180 µL of solvent to produce a diluted sample for analysis. Ten µL of Internal Standard spiking solution containing ^13^C cholic acid and ^13^C deoxycholic acid at 100 µg/mL was dispensed into 200 µL of diluted sample, the calibration standards and the blanks in LC vials and mixed by vortexing prior to analysis.

#### LC-MS/MS Analysis of Bile Samples

BA analysis was performed by LC-MS/MS. The analytes were separated using a C_18_ phase Kinetex HPLC column (15 cm × 3.0 mm ID) packed with core–shell particles of 2.6 µm (Phenomenex, Cheshire, UK). Two mobile phases were prepared: (A) water containing 0.01% formic acid/5 mM ammonium acetate, and (B) methanol containing 0.01% formic acid/5 mM ammonium acetate. Sample extracts (20 µL) were injected directly onto the HPLC column and were separated using an Agilent 1200 HPLC system using a gradient elution at a flow rate of 0.6 mL/min. The output from the HPLC was coupled to an Agilent 6410 triple quadrupole mass spectrometer (Agilent Ltd., Berkshire, UK) operated in negative ion mode with electrospray ionization.

#### LC-M/MS Data Analysis

HPLC-MS data sets were processed using Mass Hunter 3.01 software (Agilent, Manchester, UK). The acquisition time was divided into two periods 0 to 3.4 min and 3.4 to 13 min to optimize dwell time. The quantitative data were acquired using Multi Reaction Monitoring (MRM) mode. BA intensity (peak area) values were used to calculate relative BA levels in each sample from comparison with calibration standards

### Microbiome analysis

Frozen stool samples were completely thawed and DNA was isolated from approximately 1.4 ml of each homogenized sample using PSP Spin Stool DNA Plus Kit (Cat#10381102, Invitek, Berlin, Germany) according to the manufacturer's instructions. Each DNA sample was quantified by spectrophotometry (NanoDrop, ND-1000, ThermoScientific, DE).

A multiplex approach was used to identify the sequences of amplified 16S RNA genes from different stool samples. Amplification of 16S rRNA gene (V1, V2, V3 regions) was performed in triplicate using the bacterial specific primers, 27F (25-AGAGTTTGATCCTGGCTCAG-3) and 534R (5-ATTACCGCGGCTGCTGG-3). Unique sequences (“barcodes”) were incorporated into the PCR primers and amplified samples were pooled for pyrosequencing. Each sample is associated with two uniquely designed, and Roche 454 validated, 10-nucleotide barcodes. The presence of these assigned barcodes allow independent samples to be pooled together for sequencing, with subsequent bioinformatic segregation of the pyrosequencer data output.

Each 50 µL PCR reaction contained 100 ng of genomic DNA, 2× Phusion High-Fidelity PCR Master Mix with HF Buffer (Cat# M0531L, New England Biolabs, Inc., Ipswich, MA), and 0.2 µM of each primer (Integrated DNA Technologies, Coralville, IA). PCR was performed on an ABI 9700 thermocycler and included the following cycling steps: Initial denaturing at 98°C for 5 minutes followed by 40 cycles of 98°C × 30 seconds, 60°C × 45 seconds, and 72°C × 1 minute ending with a 72°C 1 × minute extension. PCR products from the extracted DNA sample were run on a 2.0% TAE agarose gel, excised and purified using QIAquick Gel Extraction Kit (Cat# 28704, Qiagen, Valencia, CA). PCR products were quantitated using Quant-iT PicoGreen dsDNA reagent (Cat # P7589, Invitrogen, Eugene, OR), normalized and pooled into 2 batches.

Pyrosequencing was performed using GS FLX Titanium series reagents (454 Life Sciences, Roche Diagnostics, Branford, CT). The barcoded and pooled amplicons were checked on an Agilent Bioanalzyer DNA7500 chip for the absence of primer-dimers, quantified with Qubit assays (Invitrogen), and diluted to 1×10^8^ molecules/uL. Emulsion PCR was set up according to the manufacturer's (Roche) protocols for the three methods, each in duplicate. Sequencing was performed using 16-region gaskets and each sample was run in two lanes. Sequencing results were analyzed with Roche software version 2.5.3, signal processing for amplicons.

Raw sequence data were processed and analyzed using the QIIME software package [31]. Reads shorter than 200 base pairs, longer than 1,000 base pairs, with more than 6 homopolymers, or with average quality less than 25 were discarded. Reverse primers were truncated from sequences and a maximum of one mismatch was allowed when matching the reverse primer. Chimeric sequences were identified and removed from the dataset using ChimeraSlayer [32]. The “open-reference” QIIME protocol was used with the UCLUST method [33] to select operational taxonomic units (OTUs). Sequences with at least 97% identity were clustered together. A representative sequence from each cluster was used to identify bacterial taxa from the May 2013 edition of the Greengenes 16S rRNA database [34]. OTUs containing less than 2 sequences were discarded, and sequences with less than 60% similarity to the Greengenes database were also discarded to remove potential contaminants from the dataset. After preprocessing, the dataset contained 360,386 sequences, of which 311,886 could be assigned to OTUs. A total of 16,834 distinct OTUs were detected among 42 samples. The fewest reads assigned to any sample was 2,461.

The OTU table was rarefied to a depth of 2,450 sequences and the resultant table was used for diversity analyses per recommended guidelines [35]. Beta-diversity was estimated using the UniFrac metric to calculate distance between samples [36] and visualized by Principle Coordinate Analysis (PCoA).

### Metformin analysis

Pharmacokinetic venous fasting plasma samples was analysed using a validated analytical method (GlaxoSmithKline, data on file).

### Statistical Methods

Analyses were performed using SAS, version 8.02 (SAS Institute, Cary, NC). Areas under the plasma-concentration time curve (AUC) PD parameters were calculated for the time intervals 0-4, 4-8 and 0-12 h (where breakfast was eaten at 0 h, lunch at 4 h and dinner at 10 h). Time-averaged, weighted-mean (WM) AUCs were calculated by dividing the area by the time interval. Repeated measures models (compound-symmetry correlation structure) were used to estimate mean fold-changes in endpoints between visits following appropriate log transformations. Missing data was not imputed. Values outside the quantifiable range were treated as censored using a likelihood-based approach. All 95% confidence intervals and associated p-values were calculated for individual sampling time points and AUCs. No adjustment has been made for multiplicity.

For the microbiome analysis, differences in relative abundance of taxa between On- and Off-metformin visits were determined by ANOVA using Subject as a blocking factor. OTUs were summarized to the Phylum level and the baseline abundance of each Phyla at Visit 1 for each patient was subtracted from later visits to represent change in Phyla representation. Changes in relative abundance were tested for correlations with patient biochemical measurements. For all statistical tests, the Benjamini-Hochberg false discovery rate adjustment [37] was used to account for the number of taxa tested in each comparison.

## Results

A summary of the baseline demographic characteristics of the subjects enrolled in this study is provided in Table 2.

**Table 2:** Disposition and Baseline Demographics of the Subjects with T2DM.

**Number of Subjects**	
Number of subjects planned, N:	12
Number of subjects entered, N:	14
Number of subjects included in All subjects (safety) population, n (%):	14 (100)
Number of subjects included in PK population, n (%):	14 (100)
Number of subjects completed, n (%):	12 (86)
Number of subjects withdrawn (consent withdrawn), n (%):	2 (14)
Number of subjects withdrawn for AE, n (%):	0 (0)
**Demographics**	
**Age (Years), Mean (SD)**	56 (5.4)
**Sex, n (%)**	
Female:	12 (86)
Male:	2 (14)
**BMI (Kg/m^2^), Mean (SD)**	30.0 (3.3)
**Height (m), Mean (SD)**	1.73 (0.08)
**Weight (Kg), Mean (SD)**	90.3 (12.2)
**HbA1c (% and mmol/mol) Mean (SD)**	7.2 (2.7)
	55.3 (5.9)
**Race, n (%)**	
African American/African Heritage	1 (7)
White – White/Caucasian/European Heritage	13 (93)

### Biochemical changes

**Figure 2:**
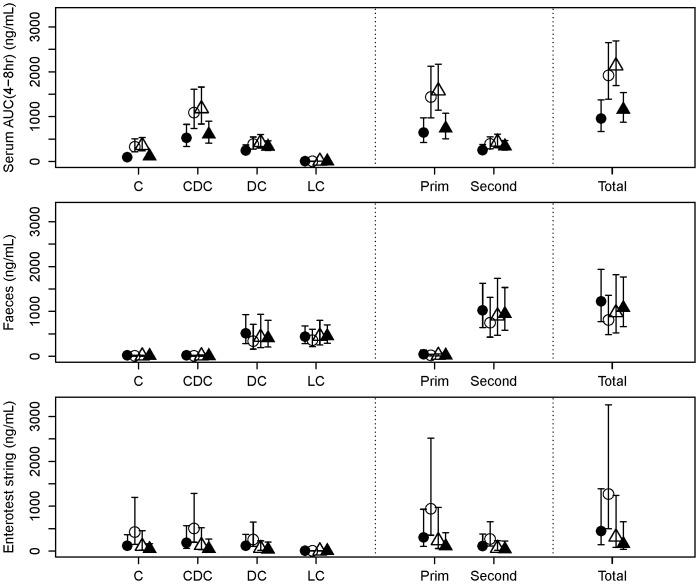
Postprandial glucose profiles. Mean plasma glucose profiles during the postprandial period of the day (left) and weighted mean AUC (± 95% confidence interval) over 0–4, 4–8 and 0–12 h (right). Data points are coded for visit number where: black circles  =  Visit 1; white circles  =  Visit 2; white triangles  =  Visit 3; black triangles  =  Visit 4.

Following metformin withdrawal at Visit 1, the 12-hour mean venous plasma glucose levels increased by ∼15% (p = 0.0006) from baseline to Visit 3 when fasting CBG had increased by ∼25%. The plasma glucose concentrations were decreased by ∼21% (p<0.0001) from Visit 3 to Visit 4 when fasting CBG had returned to baseline levels following the reintroduction of metformin. (figure 2).

**Figure 3:**
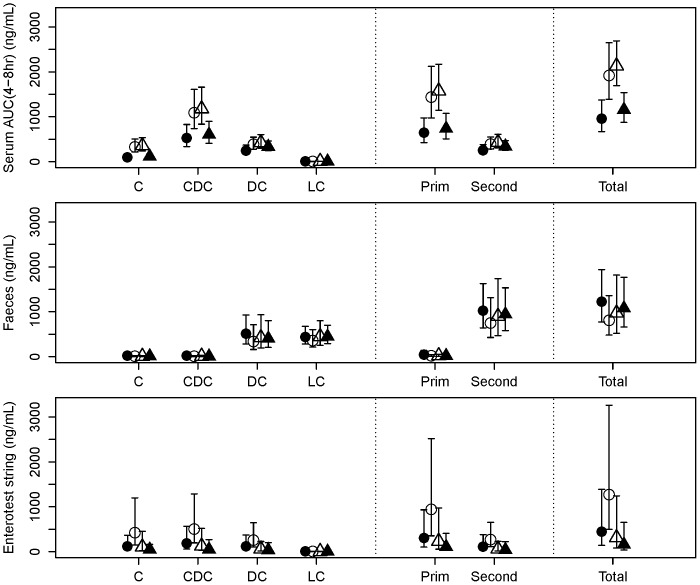
Bile acids. Bile acid concentrations (mean ± 95% confidence interval) in serum (AUC 4–8 h) (top), faeces (middle) and eluted bile from Entero-Test string (bottom). Bile acids: C  =  Cholic; CDC  =  Chenodeoxycholic; DC  =  Deoxycholic; LC  =  Lithocholic; Prim  =  Primary; Second  =  Secondary.

Seven days after the start of metformin withdrawal at Visit 1, total bile acids had increased approximately 2-fold in serum (p = 0.0012) and 3.3-fold in the EnteroTest string bile samples (p = 0.078), and decreased approximately 1.5-fold in faeces (p = 0.088) (Figure 3). Changes from baseline to Visit 3 (at which fasting CBG had increased by ∼25%) were in the same direction, but of smaller magnitude. At Visit 4, when fasting CBG had returned approximately to baseline after metformin had been reintroduced, the concentrations of bile acids in serum and faeces had returned to levels similar to baseline. The same pattern of changes was seen in both primary and secondary bile acids, but they were of slightly larger magnitude for the primary ones. In particular, between Visit 1 (baseline) and Visit 2 (7 days post-withdrawal), cholic acid plus its conjugates had increased approximately 3.2-fold in serum (p<0.0001) and 4.0-fold in the Entero-Test bile (p = 0.062), and had decreased approximately 2.4-fold in faeces (p = 0.19).

**Figure 4:**
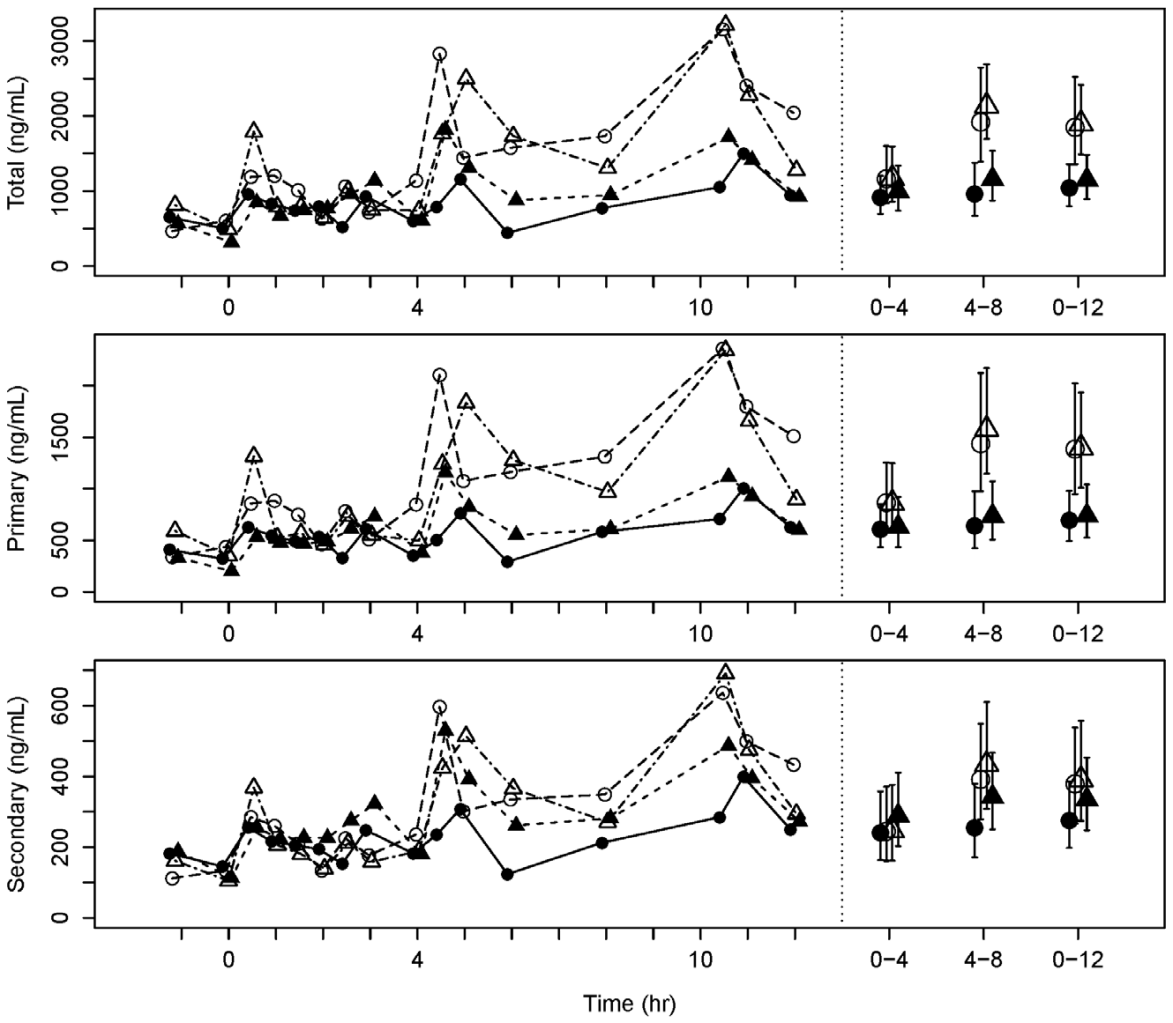
Postprandial serum bile acid profiles. Mean serum total, primary and secondary bile acid concentration profiles over 12(left) and weighted mean AUC (± 95% confidence interval) over 0–4, 4–8 and 0–12 h (right). Data point shape and color represent different visits as described in Figure 2.

Figure 4 shows the serum profiles of total, primary and secondary bile acids. It is evident that serum bile acids peaked 30–60 minutes after a meal, with distinct peaks observed after lunch and dinner on the visits when metformin had been withdrawn (Visits 2 and 3). As a result, the AUC (4–8 h) exhibited the greatest changes between visits.

**Figure 5:**
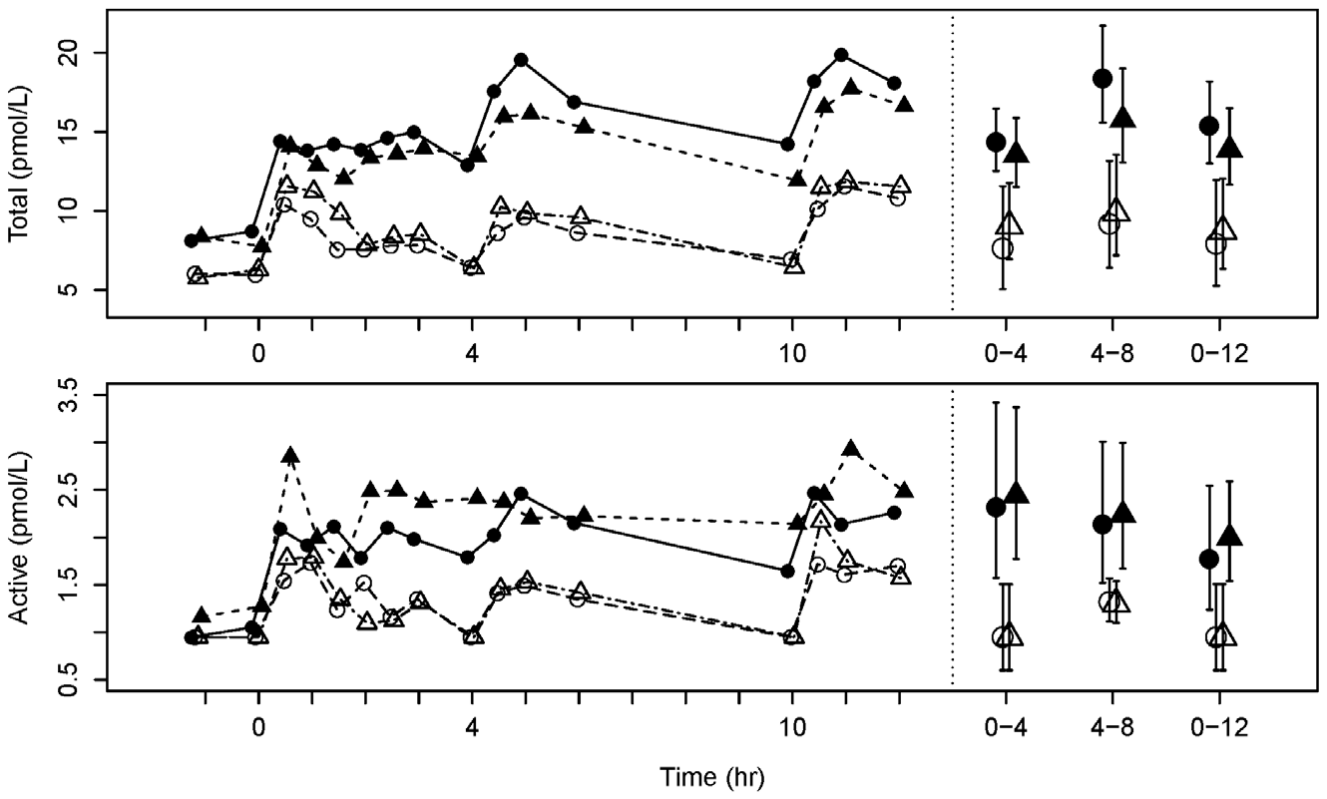
Circulating GLP-1 profiles. Mean total (top) and active (bottom) plasma GLP-1 profiles over 12 h (left) and weighted mean AUC (± 95% confidence interval) over 0–4, 4–8 and 0–12 h (right). Data point shape and color represent different visits as described in Figure 2.

**Figure 6:**
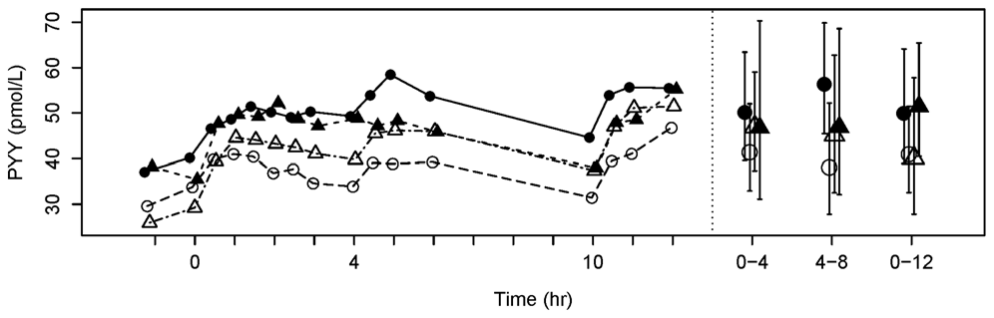
Circulating PYY profiles. Mean plasma PYY mean profiles over 12(left) and weighted mean AUC (± 95% confidence interval) over 0–4, 4–8 and 0–12 h (right). Data point shape and color represent different visits as described in Figure 2.

Mean serum concentrations of active GLP-1_7–36_ were decreased approximately 5-fold from the baseline Visit 1 to the 7-day withdrawal Visit 2 (p<0.0001), and decreased approximately 1.5-fold from the baseline Visit 1 to Visit 3 when fasting CBG had increased by ∼25% (p = 0.0030). In contrast, increases of active GLP-1 of similar magnitude were seen at Visit 4 when fasting CBG was back at baseline after metformin treatment had been resumed (p<0.0001). Changes of the mean profiles of total GLP-1 were similar, although the fold-change from baseline to Visit 2 was less marked (∼1.8-fold; p<0.0001) (figure 5). PYY showed a somewhat similar profile to GLP-1, but with a smaller fold-change from baseline Visit 1 to the 7-day withdrawal Visit 2 (∼1.3-fold decrease; p = 0.0040), and little change at Visit 3 when fasting CBG had increased by 25% (figure 6). Mean GIP concentrations changed less than 12% between any visits (all p>0.055).

### Microbiome analyses

**Figure 7:**
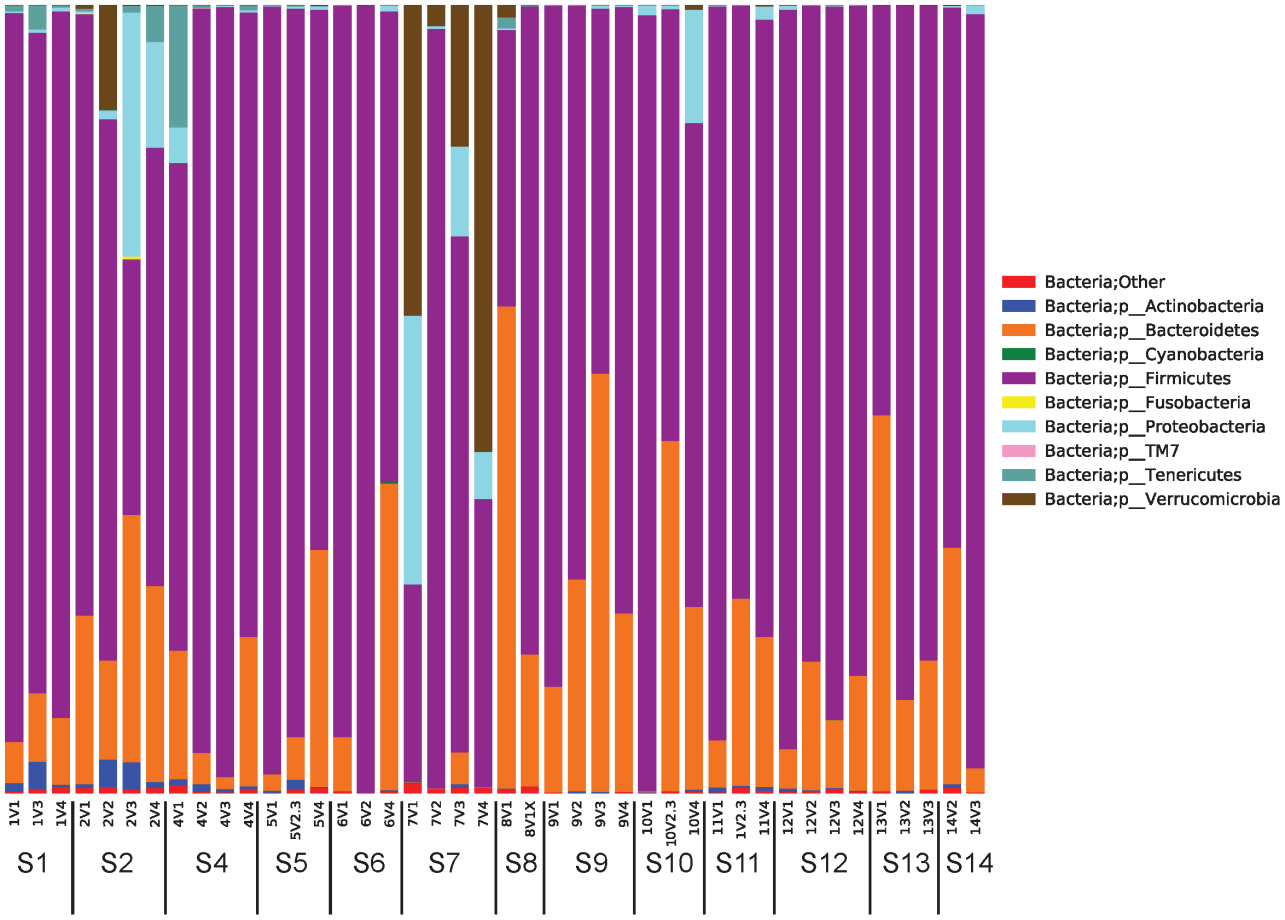
Relative abundance of microbial species across stool samples based on 16s rRNA V1–V3 region analysis. For each subject (S#), samples are ordered from first to last time points. Bacterial abundances determined at the phylum level are shown, although further analyses were completed at all intermediate taxonomic levels to genus.

**Figure 8:**
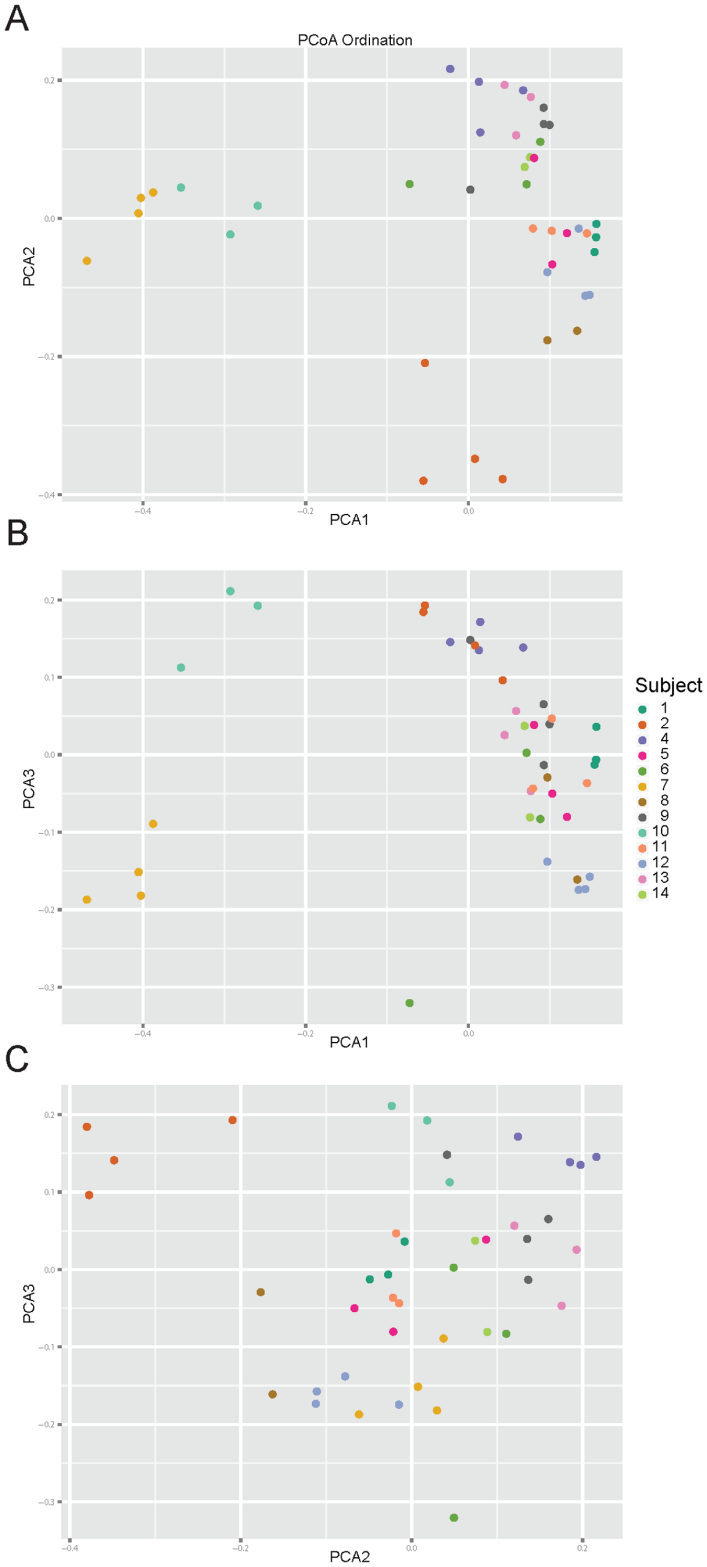
Principal coordinate analysis of microbiome beta diversity. QIIME software [31] was used to generate PCoA plots of beta (inter-sample) diversity of samples which are colored by subject. Different PCoA axes plots shown are: (a) PCA1 vs PCA2; (b) PCA3vs PCA1, and (c) PCA3 vs PCA2.

The taxa summary plots reveal heterogeneity between subjects and between visits within each subject (Figure 7). Bacterial composition of gut communities for subjects 2 and 7 were markedly different from the rest of the cohort. This observation is further supported by the sample clustering in the beta-diversity PCoA plots (Figure 8). For these reasons, subjects 2 and 7 were removed before further statistical analyses of the microbiome were completed.

**Figure 9:**
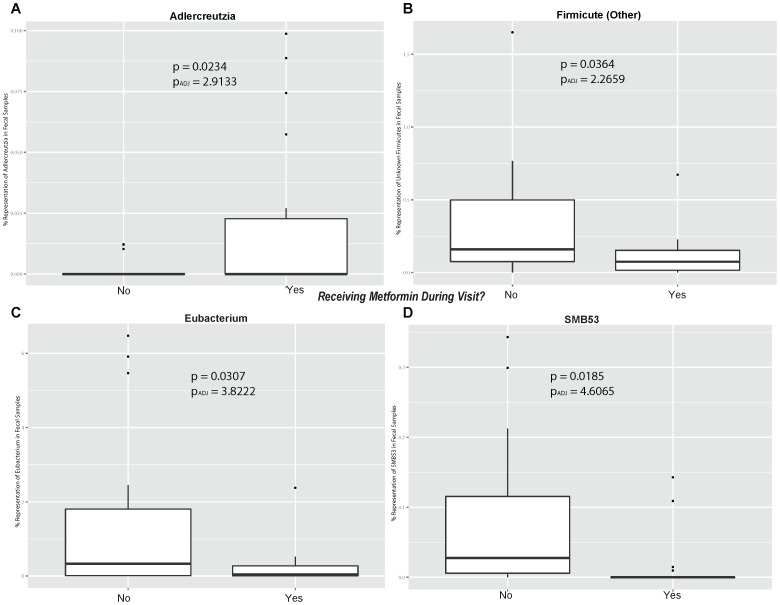
Box plots of bacterial genus On-metformin versus Off-metformin. Plots are shown for the bacterial genera (a) Adlercreutzia, (b) Firmicute (other), (c) Eubacterium and (d) SMB53. Bacterial adjusted P values used the FDR correction as described in the Methods.

In general, the beta-diversity analysis (Figure 8) shows that gut communities are patient-specific. The UniFrac distance metric calculates the overlap of phylogenetic trees between pairs of samples and the PCoA plot organizes samples so that similar communities localize near each other. Samples from the same individual cluster near each other regardless of visit number or metformin status. Because the gut communities are individual-specific, tests for treatment effects must consider the variability between subjects. Longitudinal studies of individual subjects, such as this one, enable direct evaluation of treatment effects. To determine if metformin status has an effect on taxa abundance, data from visits 1 and 4 were grouped into an On-metformin condition and data from visits 2 and 3 were grouped into an Off-metformin condition (Table 3). For each genus, one-way ANOVA was used to test for significant differences between conditions and Subject was used as a blocking factor (Figure 9A-D). Relative abundances of four genera were significantly different between On- and Off-metformin conditions, though these differences were not significant after FDR correction for the total number of genera tested in the dataset. It is possible that with a larger, independent cohort, relative abundance of these taxa may significantly predict metformin status.

**Table 3:** Gut microbiome analysis On- and Off-metformin.

Taxa		P-value	Adjusted P-value	Community Representation (%)	
Phylum	Genus			On-metformin	Off-metformin
Firmicutes	SMB53	0.0185	4.6065	0.014%	0.084%
Actinobacteria	Adlercreutzia	0.0234	2.9133	0.240%	1.534%
Firmicutes	Eubacterium	0.0307	2.5481	0.021%	0.002%
Firmicutes	Other	0.0364	2.2659	0.110%	0.347%

Adjusted P-value  =  p-value from ANOVA multiplied by number of Taxa.

**Figure 10:**
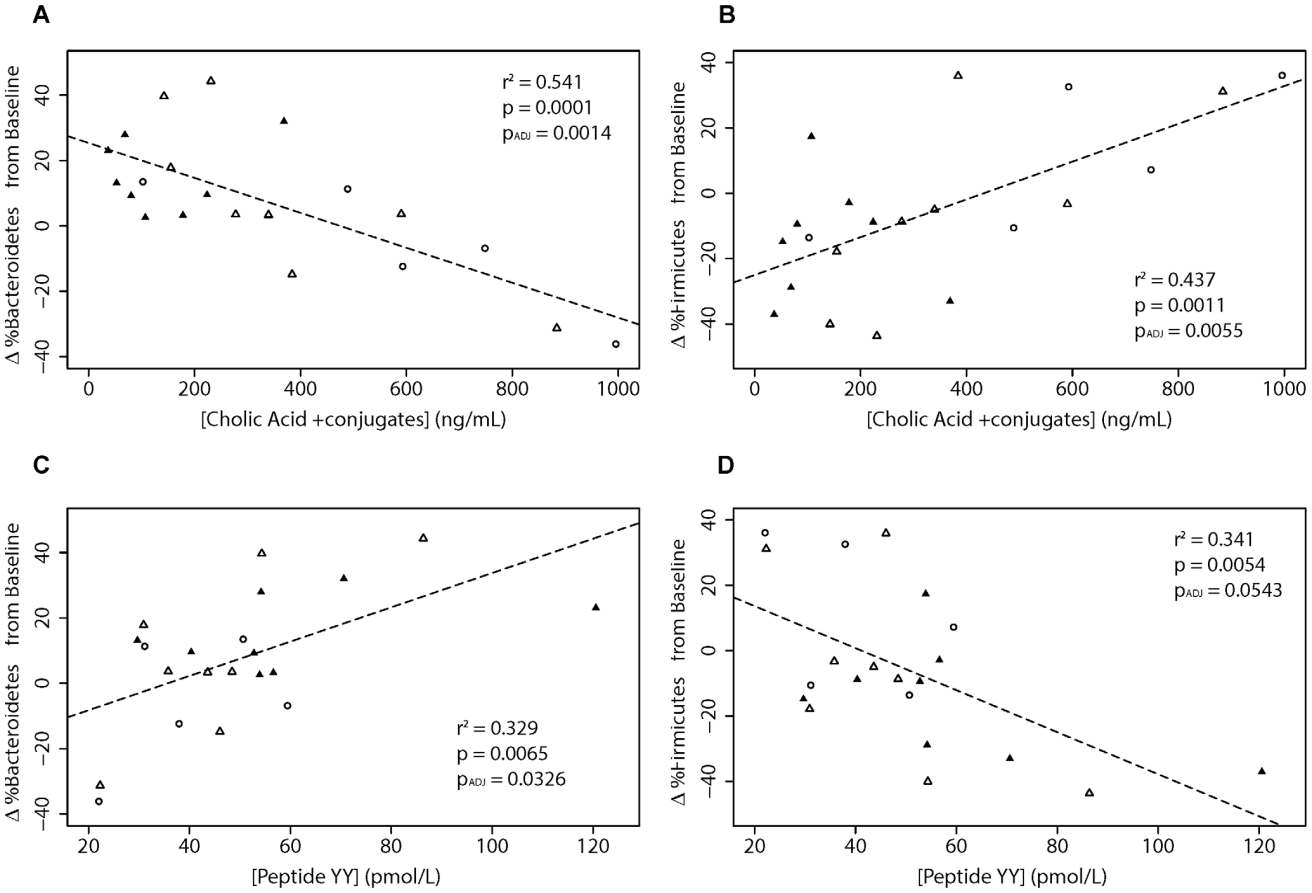
Correlations of bacterial species abundance with bile acids and PYY. Values are proportional changes in bacteria from baseline plotted for (a) Bacteriodetes vs cholic acid plus conjugates, (b) Firmicutes vs cholic acid plus conjugates, (c) Bacteriodetes vs PYY and (d) Firmicutes vs PYY. Adjusted P values used the FDR correction as described in the Methodology. Data point shape and color represent different visits as in Figure 2.

Relationships between patient biochemistry and gut communities provide insight into host-bacteria interactions. In this study, the concentration of cholic acid and conjugates (CA) in patient serum was significantly correlated with Phyla Firmicutes and Bacteroidetes abundances (Figure 10A & B). Additionally these two phyla were significantly correlated with circulating concentrations of PYY in patient sera (Figure 10 C & D). It appears that these relationships are independent of each other, because there is only a weak correlation between levels of PYY and CA in patient sera (r^2^ = 0.09, p-value = 0.55).

## Discussion

We have demonstrated that metformin has effects on bile acid metabolism, entero-endocrine hormone secretion and gut microbiome changes in patients with T2DM, challenging the concept that the glucose lowering effects of metformin are attributable solely to activation of AMPK [38], and antagonism of glucagon-mediated elevation of cAMP in the hepatocytes [39]. Furthermore, because metformin acts as a small polyvalent cation at physiological pH, after oral administration, it exhibits complex and prolonged pharmacokinetics within the gastrointestinal tract because of continual influx into, and efflux from the gut epithelial cells via the OCT-1 and other transporters [40]. Our data support the compelling evidence that indicate that metformin administered intravenously to achieve therapeutically relevant blood concentrations does not improve glucose metabolism, in contrast to oral dosing [9], [28].

First, we investigated the entero-hepatic flux of bile acids by analyzing three matrices (blood, faeces and bile eluted from the Entero-Test string) which had been collected in patients who had been sampled at baseline (On-Metformin Visit 1), after a 7 days withdrawal (Visit 2), after a variable period determined by the partial loss of fasting glycaemic control (a 25% increase of the mean CBG obtained at baseline; Visit 3), and finally after reinstatement of the usual daily dose of metformin and restitution of baseline fasting CBG values (Visit 4). Serum bile acids were the least variable of the 3 matrices tested. Total bile acids (TBA) increased significantly after metformin was withdrawn, mainly driven by changes in the primary bile acid fraction. As expected, secondary bile acids (SBA) (mainly lithocholic and deoxycholic acids) were increased in the faecal samples obtained from the same subjects. We noted that primary bile acids (PBA), and in particular chenodexoxycolic acid, were significantly elevated around lunch time (between 4–8 h). This may be due to the different composition of macronutrients in the lunch meal. Rapid changes in 7α-hydroxy- 4-cholesten-3-one (C4) synthesis have been reported as a biomarker of increased bile acid synthesis [41]. However, we did not measure C4 in our study, so we cannot differentiate between increased bile acid uptake from the ileum and increased bile acid synthesis in the liver and subsequent reuptake as an explanation for the observed increase of serum TBA when metformin was withdrawn. Our data are consistent with the report by Carter et al. [24] and suggest that metformin may inhibit the reabsorption of bile acids by altering the function of the sodium-dependent intestinal bile acid transporter.

It is intriguing to speculate that metformin activates bile acid receptors such as FXRs and TGR5 [23] by changing bile acid pools in the gut. TGR5 is expressed in non-parenchymal liver cells, in brown adipose tissue and entero-endocrine L-cells secreting GLP-1 and PYY [42]. Thus, metformin could alter GLP-1 and PYY secretion indirectly by its action on bile acid disposition in the gut. This is consistent with our data showing that withdrawal of metformin reduced total and active GLP-1 levels. The relative magnitude of the effects on active and total GLP-1 suggests that metformin may also have an inhibitory effect on the metabolizing enzyme, DPP-IV, in addition to a secretagogue action at the L cell. Enhanced L-cell secretion would have the potential for local peptide effects in the gut and more distant hormonal actions in the portal venous system and liver [43].

Secondly, we present the first evidence that the human gut microbiome profile in patients with T2DM changes when subjects are On- or Off-metformin. The gut microbiome appears to play a crucial role in the control of the host metabolism and this is an area of intense research [44]. The gut microbiota affect the metabolism of xenobiotic drugs, and different microbiomes have been associated with obesity and T2DM [45]. Our analysis of 16S RNA-derived data suggests that microbial communities are subject-specific, as we observed higher sample variability between individuals than within individuals as shown in Figures 7 and 8. When subject visit samples were pooled into categories of Off-metformin (Visits 2 and 3) or On-metformin (Visits 1 and 4), the abundances of several bacterial genera were observed to be significantly different. These associations of metformin treatment and bacterial species were not significant after multiple factor corrections, probably due to the small number of subjects in our exploratory study. However, changes in serum cholic acid, the most abundant primary biliary acid, are significantly correlated with changes in taxa representation (*Bacteroidetes* and *Firmicutes*).

While these findings need to be confirmed in larger clinical studies, the changes in the microbiome observed in our study are intriguing. *Adlercreutzia* species, which were significantly elevated in On-metformin fecal samples, are known to metabolize daidzeins (soybean isoflavonoids to equol) in the human gut [46], [47]. Dietary soybean isoflavones can favorably affect the metabolic phenotypes associated with T2DM, possibly through a mechanism where equol regulates glucose uptake in adipocytes by modulating known insulin-stimulation pathways such as peroxisome proliferator-activated receptor gamma (PPAR-gamma) mediated transcriptional activity [48]. The metabolism of daidzein into equol via microbial glucuronidation is similar across mammals [49], thus further studies involving alteration of the gut microbiome, specifically changes in *Adlercreutzia* sp., in model organisms might be useful in better understanding this aspect of metformin pharmacology.

Our study adds to the growing body of literature suggesting causal relationships between metabolic disorders and gut microbiome composition. Studies of the gut microbiome in lean and obese mice suggest that gut bacteria have the ability to impact energy homeostasis not only by influencing the efficiency of harvesting of calories from the diet, but also by altering how this harvested energy is utilized and stored [50], [51]. Early reports of a decreased proportion of Bacteroidetes in obese individuals relative to lean individuals, as well as correlations between increases in the prevalence of Bacteroidetes with weight loss produced by two different types of low-calorie diet, suggested that gut bacteria may play a role in the genesis of obesity [52]. Further animal studies support the association between dysbiosis of the gut microbiota and low grade inflammation, obesity and T2DM [53], [54] through altered gut permeability, endotoxin-mediated inflammation and insulin resistance. Our finding of significant correlations between Bacteriodetes and Firmcutes abundances and levels of cholic acid and PYY suggest mechanistic drivers behind earlier reports of the correlation between Firmicutes/Bacteriodes ratios and glycemic control in T2DM subjects [55]. The correlations between bacterial phyla and serum bile acid levels are intriguing because rats fed cholic acid show similar changes in the relative abundance of Firmicutes and Bacteroidetes [56]. While this suggests that the observed Firmicutes and Bacteroidetes abundance changes in human subjects result from fluctuations in primary bile acid concentrations, it does not preclude the possibility of a feedback loop between cholic acid and Firmicutes/Bacteroidetes ratio.

Recently, Cabreiro *et al.* showed that metformin alters the gut microbiota, disruptsmicrobial metabolic pathways and promotes longevity in the worm *C. elegans* [26]. Our study extends to humans the potentially important role that microbial-host interactions have in the pharmacology and efficacy of metformin in T2DM patients.

The main limitation of our exploratory study is the small number of subjects studied which resulted in only women completing all the study procedures. While we cannot rule out significant gender differences in the metformin effects we describe, it is our belief that these novel insights into the pharmacology of metformin justify further investigation with larger numbers of subjects, especially to determine whether there are subgroup differences that could predict enhanced or reduced efficacy or side effects of metformin.

In conclusion, we report that the pleotropic effects of metformin include alteration of the entero-hepatic recirculation of bile acids, modulation of gut microbiota and changes in gut hormones, especially GLP-1. These findings suggest that the gastrointestinal tract is an important target organ of metformin and are consistent with the evidence that oral formulations of metformin are more effective than intravenous administration.

## Supporting Information

Checklist S1 **TREND Checklist.**
(PDF)

Protocol S1 **Trial Protocol.**
(DOCX)
