# Correction: Novel Gut-Based Pharmacology of Metformin in Patients with Type 2 Diabetes Mellitus

**DOI:** 10.1371/journal.pone.0106594

**Published:** 2014-08-25

**Authors:** The PLOS ONE Staff

Figure 2 was incorrectly published as a duplicate of Figure 3. Please see the corrected Figure 2 here.

**Figure 2:**
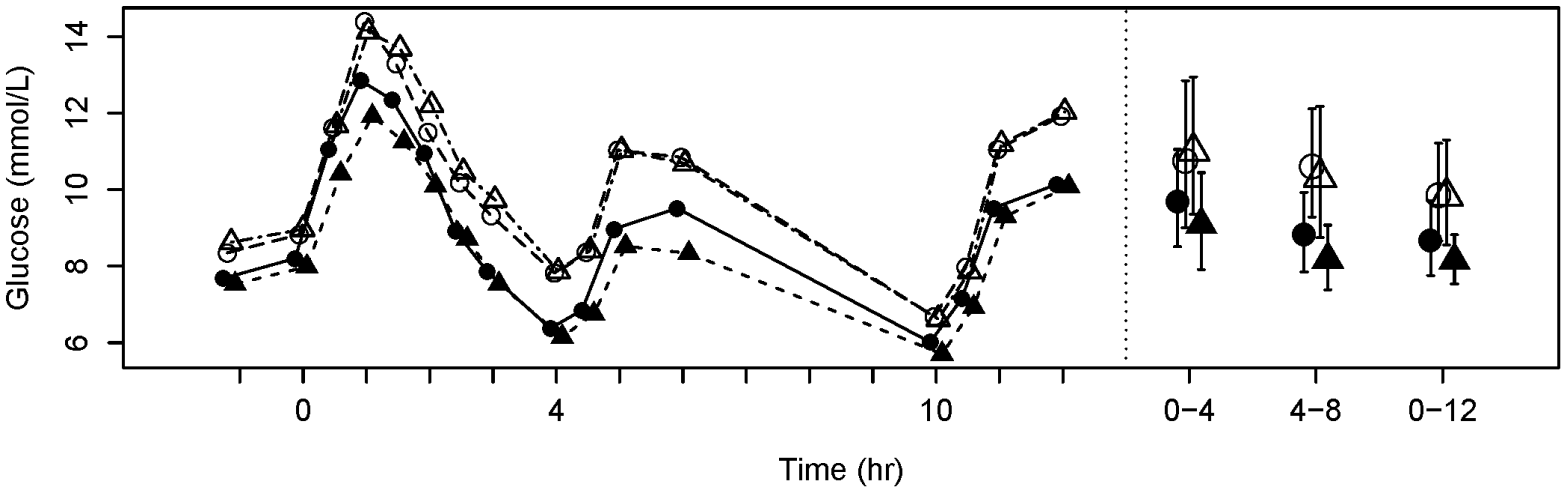
Postprandial glucose profiles. Mean plasma glucose profiles during the postprandial period of the day (left) and weighted mean AUC (± 95% confidence interval) over 0–4, 4–8 and 0–12 h (right). Data points are coded for visit number where: black circles  =  Visit 1; white circles  =  Visit 2; white triangles  =  Visit 3; black triangles  =  Visit 4.

## References

[1] Napolitano A, Miller S, Nicholls AW, Baker D, Van Horn S, et al (2014) Novel Gut-Based Pharmacology of Metformin in Patients with Type 2 Diabetes Mellitus. PLoS ONE 9(7): e100778 doi:10.1371/journal.pone.0100778 24988476 10.1371/journal.pone.0100778 PMC4079657

