# Supplementary material for: Novel Gut-Based Pharmacology of Metformin in Patients with Type 2 Diabetes Mellitus
Source: PLoS One. 2014 Jul 2;9(7):e100778. doi: 10.1371/journal.pone.0100778 (PMC4079657; doi:10.1371/journal.pone.0100778)

**Division:** Worldwide Development

**Retention Category:** GRS019

**Information Type:** Clinical Pharmacology Protocol

| **Title:** | Effect of Metformin on gut peptides , bile acids and lipid profiles in Type 2 Diabetics | |
| --- | --- | --- |
| **Compound Number:** | | None |
| **Effective Date:** | | 07-JUN-2010 |

**Description**: Metformin is a biguanide that is marketed as an oral anti-diabetic drug. Metformin treatment in concert with diet and exercise is the consensus first-line treatment for type 2 diabetes mellitus (T2DM), and therefore it will likely be an adjunct therapy for all assets in development by GSK for the treatment of T2DM. Metformin has potent effects in lowering circulating glucose concentrations, and it is believed to have additional benefits in improving macrovascular outcomes, fatty liver disease and polycystic ovarian syndrome. Its use in a significant proportion of T2DM subjects is limited by contraindications of heart failure and renal insufficiency or gastrointestinal side effects. The mechanisms underlying the glucose-lowering effect and adverse event profile of metformin are not well understood. Whilst activation of AMP kinase may be important for therapeutic effect, changes in incretin secretion and bile acid excretion have been described, but not consistently linked to its therapeutic effect or AE profile. The aim of this study is to recruit T2DM patients on prescribed metformin monotherapy to further investigate how the glucose effects are related to the alterations in bile acid absorption, incretin and lipid profiles by studying these parameters on and off the drug. This will be done in combination with frequent capillary blood glucose monitoring to ensure patient safety. This study will facilitate the development of a pharmacodynamic model that can be used by clinical teams developing non-absorbable NCEs such as iBAT inhibitors.

**Subject:** Diabetes, Metformin, Bile Acid, Incretin, Lipid Profile

**Author(s): Antonella Napolitano, Derek Nunez, Darren Robertson, Sam Miller, Andy Nicholls, Robert Dobbins**

Copyright 2010 the GlaxoSmithKline group of companies. All rights reserved. Unauthorised copying or use of this information is prohibited.

Sponsor Signatory:

| Prof Ed Bullmore VP Experimental Medicine & Head Drug Discovery |  | **Date** |
| --- | --- | --- |

Sponsor/medical monitor Information Page

**Medical Monitor and Sponsor Contact Information:**

| **Role** | Name | Day Time Phone Number | After-hours Phone/Cell/ Pager Number | Fax Number | GSK Address |
| --- | --- | --- | --- | --- | --- |
| Primary Medical Monitor | Dr Annelize Koch | 0 1223 296084 | Call +44 (0) 1223 296001. CUC security will contact the on-call Medical Monitor | 01223 296108 | GlaxoSmithKline  Addenbrooke’s Hospital  Hills Road  Cambridge  CB2 2GG, UK |
| Secondary Medical Monitor | Dr Derek Nunez | 001 919 483 2237  Mobile- 001 919-271-0008 | N/A | 001 919 315 2348 | N2.3211  GlaxoSmithKline Five Moore Drive P.O. Box 13398 RTP (Research Triangle Park) North Carolina 27709-3398 US |
| Tertiary Medical Monitor | Dr. Robert Dobbins | 001 919 483 7922 | N/A | 001-919 641 7465 | N2.3209  GlaxoSmithKline Five Moore Drive P.O. Box 13398 RTP (Research Triangle Park) North Carolina 27709-3398 US |

**Sponsor Registered Address:**

GlaxoSmithKline Research & Development Limited
980 Great West Road
Brentford
Middlesex, TW8 9GS
UK

In some countries, the clinical trial sponsor may be the local GlaxoSmithKline affiliate company (or designee). If applicable, the details of the alternative Sponsor and contact person in the territory will be provided to the relevant regulatory authority as part of the clinical trial application.

Investigator Protocol Agreement Page

I confirm agreement to conduct the study in compliance with the protocol.

- I acknowledge that I am responsible for overall study conduct. I agree to personally conduct or supervise the described clinical study.
- I agree to ensure that all associates, colleagues and employees assisting in the conduct of the study are informed about their obligations. Mechanisms are in place to ensure that site staff will receive the appropriate information throughout the study.

| Investigator Name: | Dr Antonella Napolitano | |
| --- | --- | --- |
| Investigator Address: | GlaxoSmithKline  Addenbrooke’s Hospital  Hills Road  Cambridge  CB2 2GG, UK | |
| Investigator Phone Number: | 01223 296075 | |
| Investigator Signature | | Date |

TABLE OF CONTENTS

Page

ABBREVIATIONS 2

1. Introduction 2

1.1. Background 2

1.2. Study Rationale 2

1.3. Summary of Risk Management 2

2. OBJECTIVE(S) 2

2.1. Primary 2

2.2. Secondary 2

2.3. Exploratory Objective 2

3. ENDPOINT(S) 2

3.1. Primary 2

3.2. Secondary 2

3.3. Exploratory 2

4. INVESTIGATIONAL PLAN 2

4.1. Study Design/Schematic 2

4.2. Discussion of Design 2

4.2.1. Screening visit 2

4.2.2. Study Visits 2

4.3. Treatment Assignment 2

4.3.1. Dose Adjustment/Stopping Efficacy Criteria 2

4.4. Time and Events Table 2

5. Study Population 2

5.1. Number of Subjects 2

5.2. Eligibility Criteria 2

5.2.1. Inclusion Criteria 2

5.2.2. Exclusion Criteria 2

6. Data Analysis and Statistical Considerations 2

6.1. Hypotheses and Treatment Comparisons 2

6.2. Sample Size Considerations 2

6.2.1. Sample Size Assumptions 2

6.2.2. Sample Size Sensitivity 2

6.2.3. Sample Size Re-estimation 2

6.2.4. Interim Analysis 2

6.2.4.1. Safety Analyses 2

6.2.4.2. Pharmacokinetic Analyses 2

6.2.4.3. Pharmacodynamic/Biomarker Analyses 2

7. Study Assessments and Procedures 2

7.1. Screening 2

7.1.1. Demographic/Medical History Assessments 2

7.1.2. Safety 2

7.2. Study visit 2

7.2.1. Faecal Collection 2

7.2.2. Blood Sample Collection 2

7.2.3. Bile Sample collection (EnteroTest) 2

7.2.4. Food Cue 2

7.3. Safety 2

7.4. Pharmacokinetics 2

7.4.1. Blood Sample Collection 2

7.4.2. Sample Analysis 2

7.5. Biomarker(s)/Pharmacodynamic Markers 2

7.5.1. Exploratory Biomarkers 2

8. Lifestyle And/or Dietary Restrictions 2

8.1. Meals and Dietary Restrictions 2

8.1.1. Caffeine, Alcohol, and Tobacco 2

8.2. Activity 2

9. Concomitant Medications and Non-Drug Therapies 2

9.1. Permitted Medications 2

9.2. Prohibited Medications 2

9.3. Non-Drug Therapies 2

9.4. Medical Devices 2

10. completion or early withdrawal of subjects 2

10.1. Subject Completion 2

10.2. Subject Withdrawal Criteria 2

10.3. Subject Withdrawal Procedures 2

10.3.1. Subject Withdrawal from Study 2

10.4. Treatment After the End of the Study 2

10.5. Screen and Baseline Failures 2

11. Adverse Events (AE) and Serious Adverse Events (SAE) 2

11.1. Definition of Adverse Events 2

11.2. Definition of Serious Adverse Events 2

11.3. Method of Detecting AEs and SAEs 2

11.4. Recording of AEs and SAEs 2

11.5. Evaluating AEs and SAEs 2

11.5.1. Assessment of Intensity 2

11.5.2. Assessment of Causality 2

11.6. Follow-up of AEs and SAEs 2

11.7. Prompt Reporting of SAEs to GSK 2

11.8. Regulatory Reporting Requirements For SAEs 2

12. MEDICAL DEVICES – INCIDENTS (INCLUDING MALFUNCTIONS) 2

12.1. Definitions of an Incident 2

12.2. Time Period for Detecting Medical Device Incidents 2

12.3. Documenting Medical Device Incidents 2

12.4. Follow-up of Medical Device Incidents 2

12.5. Prompt Reporting of Medical Device Incidents to GSK 2

12.6. Regulatory Reporting Requirements for Medical Devices 2

13. Study CONDUCT CONSIDERATIONS 2

13.1. Posting of Information on Clinicaltrials.gov 2

13.2. Regulatory and Ethical Considerations, Including the Informed Consent Process 2

13.2.1. Urgent Safety Measures 2

13.3. Quality Control (Study Monitoring) 2

13.4. Quality Assurance 2

13.5. Study and Site Closure 2

13.6. Records Retention 2

13.7. Provision of Study Results to Investigators, Posting to the Clinical Trials Register and Publication 2

13.8. Data Management 2

14. References 2

Appendices 2

Appendix 1:Metformin EMC entry 2

ABBREVIATIONS

| Ae | Urinary recovery of unchanged drug |
| --- | --- |
| Ae(0-x) | Urinary recovery of unchanged drug up to fixed nominal time-point x |
| Ae(0-∞) | Complete urinary recovery of unchanged drug up to time of last measurable urinary concentration |
| Ae(0-τ) | Urinary recovery over a dosing interval |
| AE | Adverse Event |
| ALT | Alanine aminotransferase (SGPT) |
| AMPK | AMP-activated protein kinase |
| ANOVA | Analysis of Variance |
| AST | Aspartate aminotransferase (SGOT) |
| AUC | Area under concentration-time curve |
| AUC(0-∞) | Area under the concentration-time curve from time zero (pre-dose) extrapolated to infinite time |
| %AUCex | Percentage of AUC(0-∞) obtained by extrapolation |
| AUC(0-x) | Area under the concentration-time curve from zero (pre-dose) to some fixed nominal time x |
| AUC(0-t) | Area under the concentration-time curve from time zero (pre-dose) to last time of quantifiable concentration within a subject across all treatments |
| AUC(0-τ) | Area under the concentration-time curve over the dosing interval |
| β-HCG | Beta-Human Chorionic Gonadotropin |
| BA | Bioavailability |
| BE | Bioequivalence |
| BMI | Body mass index |
| BP | Blood pressure |
| BPM | Beat Per Minute |
| BQL | Below the quantification limit |
| BUN | Blood urea nitrogen |
| CBC | Complete blood count |
| CI | Confidence Interval |
| CIB | Clinical Investigator’s Brochure |
| CLr | Renal clearance |
| CL | Systemic clearance of parent drug |
| CL/F | Apparent clearance following oral dosing |
| Cmax | Maximum observed concentration |
| Cmin | Minimum observed concentration |
| Cτ | Pre-dose (trough) concentration at the end of the dosing interval |
| Ct | Last observed quantifiable concentration |
| CDMP | Clinical Document Management and Publishing |
| CO_2_ | Carbon dioxide |
| CPDS | Clinical Pharmacology Data Sciences |
| CPK | Creatine phosphokinase |
| CPMS | Clinical Pharmacokinetics Modelling & Simulation |
| CPSR | Clinical Pharmacology Study Report |
| CP-RAP | Clinical Pharmacology Reporting and Analysis Plan |
| CRF | Case Report Form |
| CRO | Contract Research Organization |
| CRU | Clinical Research Unit |
| CSSO | Clinical Science and Study Operations |
| CV | Coefficient of variance |
| DB | Discovery Biometrics |
| DBP | Diastolic blood pressure |
| DDS | Drug Development Sciences |
| DILI | Drug Induced Liver Injury |
| DMPK | Drug Metabolism and Pharmacokinetics |
| DNA | Deoxyribonucleic acid |
| EDC | Electronic data capture |
| EISR | Expedited Investigator Safety Report |
| Fabs | Absolute bioavailability of drug determined following extravascular and intravascular dosing |
| FDA | Food and Drug Administration |
| Frel | Relative bioavailability of drug determined between two formulations of the same drug following similar or different extravascular route of administration |
| FSH | Follicle Stimulating Hormone |
| FTIH | First time in humans |
| GCP | Good Clinical Practice |
| GCSP | Global Clinical Safety and Pharmacovigilence |
| GGT | Gamma glutamyltransferase |
| GLP | Good Laboratory Practice |
| GLS | Geometric Least-Squares |
| GSK | GlaxoSmithKline |
| HBsAg | Hepatitis B surface antigen |
| hCG | Human chorionic gonadotropin |
| HIV | Human Immunodeficiency Virus |
| h/hr | Hour(s) |
| HR | Heart rate |
| HWE | Hardy-Weinberg Equilibrium |
| IB | Investigator’s Brochure |
| ICH | International Conference on Harmonization of Technical Requirements for Registration of Pharmaceuticals for Human Use |
| IDMC | Independent Data Monitoring Committee |
| IDSL | Integrated Data Standards Library |
| IEC | Independent Ethics Committee |
| IND | Investigational New Drug |
| IP | Investigational Product |
| IRB | Institutional Review Board |
| IU | International Unit |
| IV | Intravenous |
| Kg | Kilogram |
| λz | Terminal phase rate constant |
| L | Liter |
| LFTs | Liver function tests |
| Ln | Naperian (natural) logarithm |
| LOQ | Limit of quantification |
| LLQ | Lower limit of quantification |
| µg | Microgram |
| µL | Microliter |
| MAT | Mean absorption time |
| MCH | Mean corpuscular hemoglobin |
| MCHC | Mean corpuscular hemoglobin concentration |
| MCV | Mean corpuscular volume |
| MedDRA | Medical Dictionary for Regulatory Activities |
| Mg | Milligrams |
| mL | Milliliter |
| MRT | Mean residence time |
| MSDS | Material Safety Data Sheet |
| Msec | Milliseconds |
| NCE | New Chemical Entity |
| NQ | Non-quantifiable concentration measured as below LLQ |
| PD | Pharmacodynamic |
| PGx | Pharmacogenetics |
| PK | Pharmacokinetic |
| PSRI | Periodic Safety Reports for Investigators |
| QC | Quality control |
| QD | Once daily |
| QTcB | QT duration corrected for heart rate by Bazett’s formula |
| QTcF | QT duration corrected for heart rate by Fridericia’s formula |
| RAP | Reporting and Analysis Plan |
| RBA | Relative Bioavailability |
| RBC | Red blood cells |
| RNA | Ribonucleic acid |
| SAE | Serious adverse event(s) |
| SAS | Statistical Analysis Software |
| SD | Standard deviation |
| SGOT | Serum glutamic-oxaloacetic transaminase |
| SGPT | Serum glutamic pyruvic transaminase |
| SOP | Standard Operating Procedure |
| SPM | Study Procedures Manual |
| SUSAR | Suspected, Unexpected, Serious Adverse drug Reaction |
| T2DM | Type 2 Diabetes Mellitus |
| T | Time of last observed quantifiable concentration |
| t½ | Terminal phase half-life |
| Τ | Dosing interval |
| Tlag | Lag time before observation of drug concentrations in sampled matrix |
| Tlast | Time of last quantifiable concentration |
| Tmax | Time of occurrence of Cmax |
| ULN | Upper limit of normal |
| UK | United Kingdom |
| WBC | White blood cells |

Trademark Information

| **Trademarks of the GlaxoSmithKline group of companies** |  | **Trademarks not owned by the GlaxoSmithKline group of companies** |
| --- | --- | --- |
|  |  | Metformin |
|  |  | Entero-Test |
|  |  | WinNonlin |

# Introduction

## Background

The metabolic syndrome and its pathological sequela, type 2 diabetes mellitus (T2DM), have become major health problems across the world. The biguanide metformin is widely used as a first-line therapy for the treatment of T2DM based on the results from outcome studies such as the UKPDS [UKPDS, 1998]. In addition, the drug has recently been proposed for the treatment and/or prevention of fatty liver diseases and polycystic ovary syndrome [Glueck, 2003]. Metformin ameliorates hyperglycaemia by reducing gastrointestinal glucose absorption and hepatic glucose production and by improving glucose utilisation [Natali, 2006]. The molecular mechanisms underlying the action of metformin appear to be related to the phosphorylation of the energy sensor AMP-activated protein kinase (AMPK), which suppresses glucagon-stimulated glucose production and causes an increase in glucose uptake in muscle and in hepatic cells [Correia, 2008; Kirpichnikov, 2002]. However the pharmacology of metformin is still not fully elucidated because activation of AMPK does not explain all its metabolic effects. Indeed there have been inconsistent reports that metformin increases active GLP-1 [Mannucci, 2004], perhaps by inhibiting DPP-IV, a dipeptidyl peptidase that cleaves a number of bioactive peptides including glucose-dependent insulinotropic peptide (GIP), glucagon-like peptide (GLP-1) and peptide-tyrosine-tyrosine (PYY) secreted from enteroendocrine K and L cells in the upper and lower gut, respectively. There are also data indicating that metformin increases bile acid content of faeces [Carter, 2003] and this may result in activation of the TGR-5 receptor on enteroendocrine cells [UKPDS, 1998] and perhaps also may contribute to the gastrointestinal intolerance that is occasionally experienced by T2DM patients taking this drug.

## Study Rationale

As metformin has become first-line therapy for T2DM patients in many countries, it is important to ensure early-on in development that novel anti-diabetic NCEs work well as add-ons to this drug. Furthermore, the broad spectrum of effects of metformin that may be related to its glucose-lowering action offers the opportunity to use this drug as an investigative tool to explore the relationship between the various pharmacodynamic endpoints. The Entero-Endocrine DPU (EE-DPU) has a strategy to develop agents that act locally in the gastrointestinal tract to control blood glucose, with little or no systemic exposure. In this situation, reliable PD endpoints are essential to monitor pharmacology because systemic drug concentration measurements would not be available to track how a drug target is being activated or inhibited. In this situation, reliable PD endpoints are essential because systemic PK measurements are not available to track how a NCE target is being activated or inhibited. Therefore, a clearer understanding of metformin’s mechanism of action as it relates to glycaemic control, lipid metabolism, bile acid excretion and gastrointestinal adverse effects will enable the EnteroEndocrine (EE) project teams to design more efficient studies for the evaluation of non-absorbable NCEs targeted to receptors that are accessible from the gut lumen, including the ileal bile acid transport inhibitor.

Because of the complexity of metformin kinetics within the gut, it is proposed to follow the rise and fall of fasting blood glucose during metformin washout and re-introduction, respectively, to determine the two appropriate timepoints for more detailed investigation. As a result, subjects will be studied on 4 occasions:

1. Whilst on their usual stable dose of metformin (baseline state),
2. 7 days after stopping metformin to replicate the washout paradigm frequently used in early phase T2DM studies in GSK
3. When fasting capillary glucose has increased by 25% from the pre-metformin washout level or two weeks from the start of the wash-out period.
4. After metformin is re-introduced, when fasting capillary glucose has returned to the pre-metformin washout level (baseline state established at screening )

## Summary of Risk Management

This study will entail the withdrawal and re-introduction of metformin under closely supervised conditions. The withdrawal of metformin will be for a maximum period up to three weeks and the glucose increases projected are not expected to result in significant long-term risk for the subjects.

If subjects do not already test blood glucose at home, a glucometer, instructions on its use, and testing strips will be provided to them for capillary blood glucose (CBG) monitoring during withdrawal and reinstatement of metformin. They will be instructed to test their blood glucose twice a day, fasting before breakfast and before dinner, and at any time they are concerned that blood glucose may have risen excessively. A written diary card will be kept by each subject for recording CBG values, beginning approximately 7 days before discontinuation of metformin after baseline assessments are completed during visit 1.

Fasting CBG values >15mM or < 3.5mM must be reported to the site at once. If fasting CBG are >15mM or < 3.5 mM on any two consecutive days during the wash-out period, the subject will be discontinued and the usual dose of metformin will be reinstated, if appropriate

Subjects are required to call the study centre while not in the unit or to alert site staff while in the clinical unit:

- When they have CBG values that are >15mM
- When they have CBG values that are <3.5mM
- When they have any concerns relating to their CBG levels
- When they have rapid, unexplained changes in their blood glucose levels

Study staff will attempt to contact the subjects daily to check on the CBG values and to record any adverse events whilst the subject is at home.

Subjects will be encouraged to keep their usual lifestyle in term of diet and exercise for all duration of the study.

The Entero-Test device is simple, safe device for the collection of duodenal bile. It is well tolerated, although some subjects may feel slight nausea on removal. Some blood may be seen on the string when removed. This occurs if the string “nicks” the oesophagus on removal, this is very minor trauma that heals rapidly and is not a cause for concern.

# OBJECTIVE(S)

## Primary

To investigate the relationship between the glucose lowering action of metformin and:

- Faecal and serum bile acid concentrations
- Enteroendocrine peptide profiles including but not limited to incretins and PYY
- Lipid metabolism including but not limited to fasting lipids and prandial TGs

## Secondary

- To provide a relative estimate of the composition of bile acids in bile sampled using the EnteroTest string
- To measure sparse metformin profiles on the days when the PD endpoints are measured

## Exploratory Objective

In the event of erroneous changes in glucose profiles observed subsequent to changes in a subjects routine metformin dosing, we may screen the subject’s genome for alleles known to alter responses to metformin such as OCT1. This analysis will be performed using samples obtained under the PGx consent.

# ENDPOINT(S)

## Primary

During metformin wash out and when treatment reinstated, pharmacodynamic endpoints will include the following as data permit:

- 24h profiles of blood glucose and insulin
- Faecal and serum bile acid profiles
- Enteroendocrine peptide profiling including but not limited to tGLP-1, tGIP, and tPYY
- Serum lipid analysis including but not limited to fasting HDL and LDL cholesterol, fasting and prandial TGs, ApoA1, ApoB and ApoE

## Secondary

- Relative bile acid composition as determined by EnteroTest bile string sampling of duodenal bile.
- Sparse metformin PK profiles will be determined from plasma samples

## Exploratory

- Genetic testing for variants of metformin influx into and efflux from hepatocytes

# INVESTIGATIONAL PLAN

## Study Design/Schematic

## Discussion of Design

Because of the complexity of the entero-cellular kinetics of metformin, the period of time for investigation after metformin discontinuation or restart will be determined by monitoring the changes in fasting glucose. Thus, gastrointestinal and related markers are measured at the most informative times when there are visible changes in glucose. Subjects will be contacted daily by telephone by site staff from 7 days before Visit 1 until discharged following Visit 4.

**Visit 1** - At the first clinical unit admission (Visit 1) full PD profiles will be obtained whilst subjects are taking their usual dose of metformin.

**Visit 2 -** The first PD profiling during the metformin washout phase will be conducted 7 days after the first baseline visit to simulate the situation in early phase studies when T2DM subjects are washed off metformin for a fixed period of time.

**Visit 3 -** During the metformin washout, the site staff will continue to monitor patient progress daily and will schedule the subject for admission into the clinical unit for PD profiling (Visit 3) when the fasting CBG has increased by 25% or approximately 3 weeks has passed, whichever is sooner. If the average fasting CBG increases by the required amount within 7 days following Visit 1, then visit 3 will be eliminated and the subject will be asked to restart metformin after visit 2 is completed.

**Visit 4-** In the metformin re-start phase of the study, site staff will inform the subject when the fasting CBG has returned to the baseline level (determined at screening) and the subject will be scheduled for admission into the clinical unit for PD profiling. If the subject’s CBG has not returned to baseline level (approximate) within approximately 14-21 days, this visit will be scheduled once the subject has achieved clinically acceptable glycaemic control, in the opinion of the principal investigator.

The study will enrol approximately 16 subjects to ensure that12 fully evaluable T2DM patients complete the study. The study will consist of a screening visit and, 4 in-house study visits and a follow up visit, taking approximately 12 weeks to complete the study.

### Screening visit

The screening visit will determine a subject’s suitability to participate in the study. This visit will last approximately 4 hours and will include;

- Obtaining written informed consent
- Full medical including medical history and prior medication
- ECG
- Demographics
- Clinical laboratory blood testing including capillary blood glucose
- Vitals, alcohol breath test and urine drug screen

Once enrolled into the study, subjects will be asked to monitor CBG twice a day, fasting before breakfast and before dinner for 1 week before the first visit to establish a mean baseline fasting CBG level. The glucose values will be recorded and reviewed daily with a study coordinator by telephone.

### Study Visits

Visit 1

Within approximately 30 days of screening, eligible subjects who provide informed consent will attend the unit for the first study visit lasting approximately 30 hrs. This visit serves as a baseline session during which the following will occur, (see time and events Section 4.4 and Section 7 for details);

- Routine admission assessments
- Blood glucose diary review
- Pharmacodynamic blood sampling for entero-endocrine peptide profiling, serum bile acid and lipid profile
- Faecal collection (bile acids)
- Enterotest duodenal bile collection (bile acids)

Subjects will be admitted to the unit on the late afternoon / evening before PD profiling is due to start. They will be given a standard meal at approximately 6pm and then fasted, except water, from 10pm onwards. At approximately 12am, patients will be asked to swallow the EnteroTest, and the string will remain in situ overnight. The following morning PD blood sampling will begin, followed by the administration of a food cue to trigger gall bladder contraction, the resulting bile being collected by the string, which will be removed 1 hour later. Following initial assessments and testing, metformin will be dosed with a breakfast served at approximately 8am. Further assessments will be timed from the beginning of this meal and are detailed in Section 4.4. The assessments and procedures carried out in each of the 4 study visits will be the same.

Visit 2

Following discharge from the unit post Visit 1, patients will refrain from taking metformin. They will closely monitor their CBG each morning fasting before breakfast and before dinner, continuing to record it in a diary card. Approximately 7 days later they will return for study visit 2. Pharmacodynamic samples, faecal and bile acid collections will be repeated according to the schedule detailed in Section 4.4.

Visit 3

On discharge patients will continue to abstain from metformin and once their mean CBG increases by approximately 25%, from baseline (based on the 7 day average of fasting CBG before Visit 1) or approximately three weeks from the beginning of wash-out, they will be informed by the study co-ordinator to attend for Visit 3. Pharmacodynamic samples, faecal and bile acid collections will be repeated according to the schedule detailed in Section 4.4.

Visit 4

Upon discharge from Visit 3, subjects will resume their usual prescribed dose of metformin treatment and return to the unit for study Visit 4 once their fasting CBG returns to their baseline level (approximate) or achieve clinically acceptable gycaemic control, as determined by the principal investigator. Pharmacodynamic samples, faecal and bile acid collections will be repeated according to the schedule detailed in Section 4.4.

## Treatment Assignment

All subjects will be assigned to an identical regimen in accordance with the treatment schedule outlined in Section 4.1.

### Dose Adjustment/Stopping Efficacy Criteria

Glucose withdrawal criteria will be implemented to ensure subject safety during withdrawal of the metformin treatment. Patients will be required to measure CBG levels from the start of the study at screening until the end of the study. Patients will be withdrawn from the study if their morning fasting blood glucose concentration is above15.0 mmol/L (270mg) for 2 consecutive days.

## Time and Events Table

| Procedure | Screening  (up to 30 days prior to Day 1) |  | Study Day (each visit 1-4) | | | | | | | | | | | | | | | | | | Follow-up  (10-14 days post-last dose) |
| --- | --- | --- | --- | --- | --- | --- | --- | --- | --- | --- | --- | --- | --- | --- | --- | --- | --- | --- | --- | --- | --- |
|  |  | Day  -1 | |  | Day 1 | | | | | | | | | | | | | | | |  |
|  |  |  |  | Pre-breakfast | | 0 h | 30 min | 1 h | 2 h | | 4 h | 4 h30min | 5 h | 6 h | 8h | 9h | 10 h | 10h30min | 11hr | 12 h |  |
| Admission to Unit |  | X | |  | |  |  |  |  | |  |  |  |  |  |  |  |  |  |  |  |
| Informed Consent | X |  | |  | |  |  |  |  | |  |  |  |  |  |  |  |  |  |  |  |
| Demographics | X |  | |  | |  |  |  |  | |  |  |  |  |  |  |  |  |  |  |  |
| Full Physical Exam | X |  | |  | |  |  |  |  | |  |  |  |  |  |  |  |  |  |  |  |
| Brief Physical Exam |  | X | |  | |  |  |  |  | |  |  |  |  |  |  |  |  |  |  |  |
| ECG | X |  | |  | |  |  |  |  | |  |  |  |  |  |  |  |  |  |  | X |
| Medical/medication/ drug/alcohol history | X |  | |  | |  |  |  |  | |  |  |  |  |  |  |  |  |  |  |  |
| Vital signs | X | X | |  | |  |  |  |  | |  |  |  |  |  |  |  |  |  |  | X |
| Serum β-hCG (women) | X | X | |  | |  |  |  |  |  | |  |  |  |  |  |  |  |  |  | X |
| Prior/Con Med review | X |  | |  | |  |  |  |  |  | |  |  |  |  |  |  |  |  |  | X |
| Clinical Lab Bloods | X |  | | X | |  |  |  |  | |  |  |  |  |  |  |  |  |  |  |  |
| Entero-Test ingestion |  | X | |  | |  |  |  |  | |  |  |  |  |  |  |  |  |  |  |  |
| Drink 500ml water |  | X | |  | |  |  |  |  | |  |  |  |  |  |  |  |  |  |  |  |
| Food Cue |  |  | | X | |  |  |  |  | |  |  |  |  |  |  |  |  |  |  |  |
| Remove Bile string |  |  | | X | |  |  |  |  | |  |  |  |  |  |  |  |  |  |  |  |
| Blood Glucose (Cap) Monitoring | X | X | | X | |  |  |  |  | |  |  |  |  |  |  |  |  |  |  | X |
| Pharmacodynamic Blood Sampling^1^ |  | X | | X | | X | X | X | X | | X^4^ | X | X | X |  |  | X^4^ | X | X | X |  |
| Urine Drug/Alcohol Breath | X | X | |  | |  |  |  |  | |  |  |  |  |  |  |  |  |  |  |  |
| Faeces Collection^2^ |  | X | | 🡨-------------------------------------------------------------------------------------------------------------🡪 | | | | | | | | | | | | | | | | |  |
| Meal |  | X | |  | | X |  |  |  | | X |  |  |  |  |  | X |  |  |  |  |
| Discharge^3^ |  |  | |  | |  |  |  |  | |  |  |  |  |  |  |  |  |  | X |  |
| Outpatient Visit | X |  | |  | |  |  |  |  | |  |  |  |  |  |  |  |  |  |  | X |
| Continued | | | | | | | | | | | | | | | | | | | | | |

4.4 Time and Events Table (continued)

| 1. Sample collection times are approximate and may vary as testing dictates 2. A single or all faecal samples passed may be taken over the duration of each study visit. If the patients do not have any bowel movement during their visit, their first faecal sample passed after discharge will be collected at home and shipped back to the GSK Clinical Unit. 3. Time of discharge may vary and will not be before all assessments have been completed 4. PD blood samples scheduled to be taken at the same time as a meal will be taken pre meal |
| --- |

# Study Population

## Number of Subjects

Approximately 16 subjects will be enrolled such that approximately 12 fully evaluable subjects complete critical assessments.

If subjects prematurely discontinue the study, additional subjects may be enrolled as replacement subjects at the discretion of the Sponsor in consultation with the investigator.

## Eligibility Criteria

### Inclusion Criteria

A subject will be eligible for inclusion in this study only if all of the following criteria apply:

1. T2DM men and women between 18 –70 years of age on stable dose of metformin
2. Subjects taking stable regimens of aspirin, ACE inhibitors, beta-blockers, calcium channel blockers, thyroid replacement hormone, and HMG-CoA reductase inhibitors (statins) will be allowed if their dose regimen(s) remain constant throughout the study period
3. HBA1C >6.5% <8.5%
4. BMI from 22.5 up to 37.5 kg/m2, inclusive
5. AST, ALT, alkaline phosphatase and bilirubin ≤ 3xULN
6. Has a normal ECG as determined by unit physician.
7. Capable of giving written informed consent, which includes compliance with the requirements and restrictions listed in the consent form.

### Exclusion Criteria

A subject will not be eligible for inclusion in this study if any of the following criteria apply:

- Medically unable or unwilling to discontinue current metformin therapy for as required by the protocol and remain off medication until the completion of Visit 3
- Past or present disease (other than T2DM) as judged by the Investigator, which may affect the outcome of this study. These diseases include, but are not limited to, cardiovascular disease, malignancy, hepatic disease, renal disease, haematological disease, neurological disease and endocrine disease.
- Subject is currently on bile acid sequestrant therapy.
- Gastrointestinal surgery or disease that would compromise the use of the EnteroTest

1. Fasting triglycerides >450mg/dL (>5.1 mmol/L)
2. Current or chronic history of liver disease, or known hepatic or biliary abnormalities (with the exception of Gilbert's syndrome or asymptomatic gallstones).
3. A positive pre-study drug/alcohol screen.
4. History of regular alcohol consumption within 6 months of the study defined as:

- An average weekly intake of >21 units for males or >14 units for females. One unit is equivalent to 8 g of alcohol: a half-pint (~240 ml) of beer, 1 glass (125 ml) of wine or 1 (25 ml) measure of spirits.

1. Use of prescription or non-prescription drugs, including vitamins, herbal and dietary supplements (including St John’s Wort) within 7 days (or 14 days if the drug is a potential enzyme inducer) or 5 half-lives (whichever is longer) prior to the first dose of study medication, unless in the opinion of the Investigator and GSK Medical Monitor the medication will not interfere with the study procedures or compromise subject safety.
2. Pregnant females as determined by positive (serum or urine) hCG test at screening or prior to dosing.
3. Lactating females.
4. Unwillingness or inability to follow the procedures outlined in the protocol.
5. Subject is mentally or legally incapacitated or unable to provide informed consent

# Data Analysis and Statistical Considerations

## Hypotheses and Treatment Comparisons

**Precision Estimation**

This study is designed to estimate the impact of temporary cessation of metformin treatment on faecal and serum bile acid concentrations, entero-endocrine peptide profiles and lipid metabolism. No formal hypotheses will be tested. Point estimates and corresponding 95% confidence intervals (CI) will be constructed for the mean levels of these parameters, their changes over time, and for the correlations between them and blood glucose and insulin.

## Sample Size Considerations

### Sample Size Assumptions

The sample size is based on feasibility. For any identified biomarker to be of practical use in future early phase studies it will have to be sufficiently sensitive that it can provide useful results in cohorts of approximately this size.

### Sample Size Sensitivity

The expected width of 95% CI for group means with 12 subjects is +/–0.62SD_b_ where SD_b_ is the between-subject standard deviation of the quantity of interest.

A sample size of 12 subjects is expected to detect “significant” correlations (i.e. different from zero) when the true correlation is greater than 0.56 in magnitude. Expected 95% CIs for a range of correlations are given in the table below.

| **Correlation** | **Expected 95% Confidence Interval** |
| --- | --- |
| 0.6 | (0.06, 0.87) |
| 0.7 | (0.23, 0.90) |
| 0.8 | (0.43, 0.94) |
| 0.9 | (0.68, 0.97) |

### Sample Size Re-estimation

No sample size re-estimation will be performed.

### Interim Analysis

No interim analysis is planned.

#### Safety Analyses

Safety data will be presented in tabular and/or graphical format and summarised descriptively.

#### Pharmacokinetic Analyses

Trough concentrations of metformin in plasma will be measured and pharmacokinetic analysis will be the responsibility of the Clinical Pharmacology Modeling and Simulation Department, GlaxoSmithKline. Plasma metformin concentration data will be analyzed by non-compartmental methods with WinNonlin Professional Version 5.2 or higher.

#### Pharmacodynamic/Biomarker Analyses

Bile acid measurements will be the responsibility of Investigative Preclinical Toxicology group within GlaxoSmithKline Ltd. Qualitative and quantitative measurements will be undertaken using a high performance liquid chromatography – mass spectrometry (LC-MS) based method. For quantitative measurements data will be obtain from comparison to authentic standards.

Bile acid quantitative measurements from blood serum and faeces will be presented in graphical and/or tabular form and will be summarised descriptively. All qualitative bile acid data will be presented in a tabular form and summarised descriptively. All data will be stored in the Archives of GlaxoSmithKline Pharmaceuticals, R&D.

Statistical analysis of the bile acid data and other primary and secondary endpoints will be the responsibility of Discovery Analytics, GlaxoSmithKline. Statistical analysis will include repeated measures models to estimate mean changes in endpoints over time (adjusted for any missing data), following appropriate transformations (e.g. log). Estimates of correlations between endpoints, and between changes in endpoints will also be produced, either non-parametric (Spearman rank) or from examining the variance-covariance matrices from multivariate repeated-measures models. Changes are expected to be largest between visits 1 and 3, so differences over this period will be investigated primarily.

# Study Assessments and Procedures

This section lists the parameters of each planned study assessment. The exact timing of each assessment is listed in the Time and Events Table (Section 4.4). Detailed procedures for obtaining each assessment are provided in the Study Procedures Manual (SPM). Whenever vital signs and blood draws are scheduled for the same nominal time, the assessments should occur in the following order: vital signs, blood draws. The timing of the assessments should allow the blood draw to occur at the exact nominal time.

The timing and number of planned study assessments, including safety, pharmacokinetic, pharmacodynamic/biomarker or others assessments may be altered during the course of the study based on newly available data to ensure appropriate monitoring. The change in timing or addition of time points for any planned study assessments must be approved and documented by GSK, but this will not constitute a protocol amendment. The IEC will be informed of any safety issues that require alteration of the safety monitoring scheme. No more than 500 mL of blood will be collected over the duration of the study, including any extra assessments that may be required.

## Screening

Volunteers will be invited to attend a consent talk at the Clinical Unit Cambridge (CUC) and, if they decide to participate in the study, they will be screened to check whether they are eligible for the study. Screening will take place up to 30 days inclusive before a volunteer is admitted to the unit for the start of the study treatment period. The screening visit will take approximately 4 hours, during which subjects will be consented and undergo a medical examination - which will include a medical history, assessment of vital signs (blood pressure and pulse rate), ECG, a urine drug screen for drugs of abuse and alcohol breath test.

### Demographic/Medical History Assessments

The following demographic parameters will be captured: date of birth, gender, race and ethnicity.

Medical/medication/alcohol history will be assessed as related to the eligibility criteria listed in Section 5.2.

### Safety

Planned time points for all safety assessments are listed in the Time and Events Table (Section4 5). Additional time points for safety tests such as vital signs, physical exams and laboratory safety tests may be added during the course of the study based on newly available data to ensure appropriate safety monitoring.

Physical Exams

- A complete physical examination will include assessments of the head, eyes, ears, nose, throat, skin, thyroid, neurological, lungs, cardiovascular, abdomen (liver and spleen), lymph nodes and extremities. Weight will also be measured and recorded.

Vital Signs

- Vital sign measurements will include systolic and diastolic blood pressure and pulse rate.

Clinical Laboratory Assessments

Hematology, clinical chemistry, urinalysis and additional parameters to be tested are listed below:

| **Hematology** | | |
| --- | --- | --- |
| Platelet Count | *RBC Indices*: | *Automated WBC Differential:* |
| RBC Count | MCV | Neutrophils |
| WBC Count (absolute) | MCH | Lymphocytes |
| Hemoglobin | MCHC | Monocytes |
| Hematocrit |  | Eosinophils |
|  |  | Basophils |

| **Clinical Chemistry** | | | |
| --- | --- | --- | --- |
| BUN | Potassium | AST (SGOT) | Total and direct bilirubin |
| Creatinine clearance | Chloride | ALT (SGPT) | Albumin |
| Glucose | Total CO_2_ | GGT | Total Protein |
| Sodium | Calcium | Alkaline phosphatase | apolipoproteins |
| HDL cholesterol | LDL cholesterol | Fasting triglycerides | Total Cholesterol |
| 24hr triglyceride profile |  |  |  |

| **Other screening tests** |
| --- |
| HbA1c |
| Fasting lipid profile |
| FSH and oestradiol (as needed in women of non-child bearing potential only) |
| Alcohol breath, urine drug screen (to include at minimum: amphetamines, barbiturates, cocaine, opiates, cannabinoids and benzodiazepines). |

## Study visit

### Faecal Collection

One or all faecal samples passed during each study visit will be collected, weighed and stored prior to shipment to GSK for analysis for faecal bile acid profiles. In the event that subjects do not pass any faeces while in the unit, the first stool sample passed after discharge will be collected at home and shipped back to CUC-GSK.

### Blood Sample Collection

Blood samples for pharmacokinetic analysis of metformin will be collected at the time points indicated in Section 4.4, Time and Events Table. The actual date and time of each blood sample collection will be recorded. The timing of PK samples may be altered and/or PK samples may be obtained at additional time points to ensure thorough PK monitoring. Details of PK blood sample collection (including volume to be collected), processing, storage and shipping procedures are provided in the Study Procedures Manual (SPM).

### Bile Sample collection (EnteroTest)


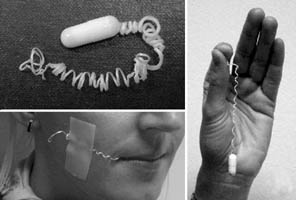


The Entero-Test allows us to sample duodenal bile. It takes the form of a capsule and has dimensions of approximately 2 cm by 0.5 cm. The subject is given approximately 500ml of water and asked to swallow the capsule.

Once swallowed the string will makes its way through the digestive tract until in position at around 4 hours after ingestion. A food cue is given to encourage the release of bile onto the string. It entails showing the volunteer images of appetising foods and asking them to imaging eating them for approximately 60 seconds. This is followed by releasing orange zest scent under the nostrils and giving a food morsel. These three elements make up the cue. After completing the food cue the at a pre determined time point Entero-Test should be withdrawn by simply pulling the string out of the subject. This should take no more than 30 seconds.

At that point, the string should be visually analysed for signs of bile (yellow staining present) or should have a pH test performed if the string is not stained. The pH test is included in the Entero-Test packaging and comes with a colour indicator.

Duodenal bile samples will be collected as described in the Time and Events Table (Section 4.4) according to the following schedule:

- Entero-Test capsule swallowed approximately 6 hrs after evening meal on Day 1. Entero-Test withdrawn 8 hrs later on Day 2.
- The gall bladder will be emptied by offering a food stimulus approximately 1 hour prior to withdrawal.
- String samples will be prepared per the SPM and shipped to GSK for analysis

### Food Cue

Exposure to food cues may include:

- Images of high calorie / appetising food
- Scent of orange zest
- The sight and smell of recently cooked, highly appetising food
- Sham feeding - a subject will be given a morsel of high fat food to chew and will be asked to remove the food from their mouth prior to swallowing.
- A morsel of high fat food for the subject to ingest

These food cues may be used in combination or separately at the time points identified in Section 4.4 (Time and Events Table). The decision of which cues will be used and when are dependent on the subject and any dietary restrictions they may have.

## Safety

Planned timepoints for all safety assessments are listed in the Time and Events Table (Section 4.4). Additional time points for safety tests (such as vital signs, physical exams and laboratory safety tests) may be added during the course of the study based on newly available data to ensure appropriate safety monitoring. Samples taken during study visits will be taken following a period of overnight fasting.

Physical Exams

- A complete physical examination will include assessments of the head, eyes, ears, nose, throat, skin, thyroid, neurological, lungs, cardiovascular, abdomen (liver and spleen), lymph nodes and extremities. Height and weight will also be measured and recorded.
- A brief physical examination will include assessments of the skin, lungs, cardiovascular system, and abdomen (liver and spleen).

Vital Signs

- Vital sign measurements will include systolic and diastolic blood pressure and pulse rate.

## Pharmacokinetics

### Blood Sample Collection

Blood samples for pharmacokinetic analysis of metformin will be collected at the time points indicated in Section 4.4, Time and Events Table. The actual date and time of each blood sample collection will be recorded. The timing of PK samples may be altered and/or PK samples may be obtained at additional time points to ensure thorough PK monitoring.

Details of PK blood sample collection (including volume to be collected), processing, storage and shipping procedures are provided in the Study Procedures Manual (SPM).

### Sample Analysis

Plasma analysis will be performed under the management of Worldwide Bioanalysis, DMPK, GlaxoSmithKline. Concentrations of metformin will be determined in plasma samples using the currently approved analytical methodology. Raw data will be stored in the GLP Archives, GlaxoSmithKline.

## Biomarker(s)/Pharmacodynamic Markers

### Exploratory Biomarkers

With the subject’s consent, blood samples will be collected during this study and may be used for the purposes of measuring novel biomarkers to identify factors that may influence T2DM and related metabolic conditions such as dyslipidemia and obesity , as well as the biological and clinical responses to metformin. If relevant, this approach will be extended to include the identification of biomarkers associated with adverse events.

Samples will be collected at the timepoints indicated in Section 4.4. The timing of the collections may be adjusted on the basis of emerging PK or PD data from this study or other new information in order to ensure optimal evaluation of the PD endpoints.

- Novel candidate biomarkers and subsequently discovered biomarkers of the biological response associated with T2DM or medically related conditions and/or the action of metformin identified by application of peptide analysis of plasma samples.
- Metabolomic analysis of blood samples, initially providing a quantitative measurement of bile acids from serum samples.
- Quantitative measurement of bile acids from faeces.
- Qualitative measurement of bile acids from the Entero-Test.

Details of the biomarker sample collection (including quantity of sample to be collected), processing, storage and shipping procedures are to be provided in the Study Procedures Manual (SPM).

All samples may be retained for a maximum of 15 years after the last subject completes the trial.

# Lifestyle And/or Dietary Restrictions

## Meals and Dietary Restrictions

Subjects will be encouraged to maintain their usual diet as well as their exercise routine.

### Caffeine, Alcohol, and Tobacco

- During each dosing session, subjects will abstain from ingesting caffeine- or xanthine-containing products (e.g. coffee, tea, cola drinks, chocolate) for 24 hours prior to the start of dosing until collection of the final pharmacokinetic and or pharmacodynamic sample during each session.
- During each dosing session, subjects will abstain from alcohol for 24 hours prior to the start of dosing until collection of the final pharmacokinetic and or pharmacodynamic sample during each session.
- Subjects who use tobacco products will be instructed that use of nicotine-containing products (including nicotine patches) will not be permitted while they are in the Clinical Unit.

## Activity

When at home, subjects should maintain their usual activities from the time they sign the informed consent form to the final follow-up visit. They will abstain from strenuous exercise for 48 hours prior to each blood collection for clinical laboratory tests. Subjects may participate in light recreational activities during studies (e.g., watch television, read*).*

# Concomitant Medications and Non-Drug Therapies

## Permitted Medications

Paracetamol at doses of ≤ 2 grams/day is permitted as well as Ibuprophen ≤ 1200 mg/day. Other concomitant medication may be considered on a case by case basis by the GSK Medical Monitor.

Stable regimens of aspirin, ACE inhibitors, beta-blockers, calcium channel blockers, thyroid replacement hormone, and HMG-CoA reductase inhibitors (statins) will be allowed if their dose regimen(s) remain constant throughout the study period. Statins are not expected to alter bile acid excretion directly, but their effects on cholesterol metabolism may indirectly reduce the concentration of bile acids in faeces.

## Prohibited Medications

Subjects must abstain from taking prescription or non-prescription drugs (including vitamins and dietary or herbal supplements), within 7 days (or 14 days if the drug is a potential enzyme inducer) or 5 half-lives (whichever is longer) prior to the first dose of study medication until completion of the follow-up visit, unless in the opinion of the Investigator and sponsor the medication will not interfere with the study.

Paracetamol should not be used in patients with acute viral hepatitis.

## Non-Drug Therapies

Subjects must abstain from taking any vitamins, herbal and dietary supplements within 7 days (or 14 days if the drug is a potential enzyme inducer) or 5 half-lives (whichever is longer) prior to the first dose of study medication until completion of the follow-up visit, unless in the opinion of the Investigator and sponsor the medication will not interfere with the study.

## Medical Devices

There are no GSK manufactured medical devices (or devices manufactured for GSK by a third party) provided for use in this study

There are however other medical devices (not manufactured by or for GSK) provided for use in this study are the EnteroTest (HDC Corporation (Milpitas, USA).

Instructions for medical device use are provided in the SPM.

# completion or early withdrawal of subjects

## Subject Completion

A completed subject is one who has completed all phases of the study including the follow-up visit.

The end of the study is defined as the last subject’s last visit, in this case the follow-up visit.

## Subject Withdrawal Criteria

A subject may withdraw from the study at any time at his/her own request, or may be withdrawn at any time at the discretion of the investigator for safety, behavioural or administrative reasons.

## Subject Withdrawal Procedures

Follow-up procedures will be conducted when a subject is withdrawn or withdraws from the study.

### Subject Withdrawal from Study

A subject may withdraw from the study at any time at their own request, or they may be withdrawn at any time based on general safety and tolerability.

If a subject is prematurely discontinued from participation in the study for any reason, the investigator will make every effort to perform the following evaluations: physical examination, vital signs (blood pressure and heart rate), clinical chemistry, haematology, urinalysis, serum/urine pregnancy test (if applicable) and AE assessment. These data will be recorded, as they comprise an essential evaluation that needs to be done before discharging any subject from the study. The reason for withdrawal or failure to provide a reason must be documented by the investigator on the Completion/Withdrawal Section of the Case Report Form (CRF).

Subjects who prematurely discontinue the study may be replaced; this will be evaluated on a case by case basis.

## Treatment After the End of the Study

Subjects will not receive any additional treatment after completion of the study because they will return to their previously prescribed metformin medication.

## Screen and Baseline Failures

Data for screen and baseline failures will not be collected.

# Adverse Events (AE) and Serious Adverse Events (SAE)

The investigator or site staff are responsible for detecting, documenting and reporting events that meet the definition of an AE or SAE.

AEs will be collected from the start of the study and until the follow-up contact. Medical occurrences that begin prior to the start of investigational product but after obtaining informed consent may be recorded on the Medical History/Current Medical Conditions CRF.

SAEs will be collected over the same time period as stated above for AEs. However, any SAEs assessed as related to study participation (e.g. investigational product, protocol-mandated procedures, invasive tests, or change in existing therapy) or related to a GSK concomitant medication will be recorded from the time a subject consents to participate in the study up to and including any follow-up contact. All SAEs will be recorded and reported to GSK within 24 hours, as indicated in Section 11.7.

Investigators are not obligated to actively seek AEs or SAEs in former study participants. However, if the investigator learns of any SAE, including a death, at any time after a subject has been discharged from the study, and he/she considers the event reasonably related to the investigational product or study participation, the investigator would promptly notify GSK.

## Definition of Adverse Events

An AE is any untoward medical occurrence in a patient or clinical investigation subject, temporally associated with the use of a medicinal product, whether or not considered related to the medicinal product.

Note: An AE can therefore be any unfavourable and unintended sign (including an abnormal laboratory finding), symptom, or disease (new or exacerbated) temporally associated with the use of a medicinal product.

Events meeting the definition of an AE **include**:

- Any abnormal laboratory test results (haematology, clinical chemistry, or urinalysis) or other safety assessments (e.g.,, radiological scans, vital signs measurements), including those that worsen from baseline, and felt to be clinically significant in the medical and scientific judgement of the investigator.
- Exacerbation of a chronic or intermittent pre-existing condition including either an increase in frequency and/or intensity of the condition.
- New conditions detected or diagnosed after investigational product administration even though it may have been present prior to the start of the study.
- Signs, symptoms, or the clinical sequelae of a suspected interaction.
- Signs, symptoms, or the clinical sequelae of a suspected overdose of either investigational product or a concomitant medication (overdose per se will not be reported as an AE/SAE).
- "Lack of efficacy" or "failure of expected pharmacological action" per se will not be reported as an AE or SAE. However, the signs and symptoms and/or clinical sequelae resulting from lack of efficacy will be reported if they fulfil the definition of an AE or SAE.
- The signs and symptoms and/or clinical sequelae resulting from lack of efficacy will be reported if they fulfil the definition of an AE or SAE. Also, "lack of efficacy" or "failure of expected pharmacological action" also constitutes an AE or SAE.

Events that **do not** meet the definition of an AE include:

- Any clinically significant abnormal laboratory findings or other abnormal safety assessments that are associated with the underlying disease, unless judged by the investigator to be more severe than expected for the subject’s condition.
- The disease/disorder being studied, or expected progression, signs, or symptoms of the disease/disorder being studied, unless more severe than expected for the subject’s condition.
- Medical or surgical procedure (e.g., endoscopy, appendectomy); the condition that leads to the procedure is an AE.
- Situations where an untoward medical occurrence did not occur (social and/or convenience admission to a hospital).
- Anticipated day-to-day fluctuations of pre-existing disease(s) or condition(s) present or detected at the start of the study that do not worsen.

## Definition of Serious Adverse Events

If an event is not an AE per Section 11.1, then it cannot be an SAE even if serious conditions are met (e.g., hospitalization for signs/symptoms of the disease under study, death due to progression of disease, etc).

An SAE is any untoward medical occurrence that, at any dose:

1. Results in death
2. Is life-threatening

NOTE: The term 'life-threatening' in the definition of 'serious' refers to an event in which the subject was at risk of death at the time of the event. It does not refer to an event, which hypothetically might have caused death, if it were more severe.

1. Requires hospitalization or prolongation of existing hospitalisation

NOTE: In general, hospitalisation signifies that the subject has been detained (usually involving at least an overnight stay) at the hospital or emergency ward for observation and/or treatment that would not have been appropriate in the physician’s office or out‑patient setting. Complications that occur during hospitalisation are AEs. If a complication prolongs hospitalisation or fulfils any other serious criteria, the event is serious. When in doubt as to whether “hospitalisation” occurred or was necessary, the AE should be considered serious.

Hospitalization for elective treatment of a pre-existing condition that did not worsen from baseline is not considered an AE.

1. Results in disability/incapacity, or

NOTE: The term disability means a substantial disruption of a person’s ability to conduct normal life functions. This definition is not intended to include experiences of relatively minor medical significance such as uncomplicated headache, nausea, vomiting, diarrhea, influenza, and accidental trauma (e.g. sprained ankle) which may interfere or prevent everyday life functions but do not constitute a substantial disruption.

1. Is a congenital anomaly/birth defect
2. Medical or scientific judgment should be exercised in deciding whether reporting is appropriate in other situations, such as important medical events that may not be immediately life-threatening or result in death or hospitalization but may jeopardize the subject or may require medical or surgical intervention to prevent one of the other outcomes listed in the above definition. These should also be considered serious. Examples of such events are invasive or malignant cancers, intensive treatment in an emergency room or at home for allergic bronchospasm, blood dyscrasias or convulsions that do not result in hospitalization, or development of drug dependency or drug abuse.
3. Is associated with liver injury **and** impaired liver function defined as:

- ALT ≥ 3xULN, and
- total bilirubin ≥ 2xULN or INR > 1.5.

**NOTES:**

Bilirubin fractionation should be performed if testing is available. If fractionation is unavailable, urinary bilirubin is to be measured via dipstick (a measurement of direct bilirubin, which would suggest liver injury).

INR measurement is not required; if measured, the threshold value stated will not apply to patients receiving anticoagulants. If INR measurement is obtained, the value is to be recorded on the SAE form.

## Method of Detecting AEs and SAEs

Care will be taken not to introduce bias when detecting AEs and/or SAEs. Open-ended and non-leading verbal questioning of the subject is the preferred method to inquire about AE occurrence. Appropriate questions include:

Care will be taken not to introduce bias when detecting AEs and/or SAEs. Open-ended and non-leading verbal questioning of the subject is the preferred method to inquire about AE occurrence. Appropriate questions include:

- “How are you feeling?”
- “Have you had any (other) medical problems since your last visit/contact?”
- “Have you taken any new medicines, other than those provided in this study, since your last visit/contact?”

## Recording of AEs and SAEs

When an AE/SAE occurs, it is the responsibility of the investigator to review all documentation (e.g., hospital progress notes, laboratory, and diagnostics reports) relative to the event. The investigator will then record all relevant information regarding an AE/SAE in the appropriate data collection tool.

It is not acceptable for the investigator to send photocopies of the subject’s medical records to GSK in lieu of completion of the GSK, AE/SAE data collection tool. However, there may be instances when copies of medical records for certain cases are requested by GSK. In this instance, all subject identifiers, with the exception of the subject number, will be blinded on the copies of the medical records prior to submission of to GSK.

The investigator will attempt to establish a diagnosis of the event based on signs, symptoms, and/or other clinical information. In such cases, the diagnosis will be documented as the AE/SAE and not the individual signs/symptoms.

Subject-completed health outcomes questionnaires and the collection of AE data are independent components of the study. Responses to each question in the health outcomes questionnaire will be treated in accordance with standard scoring and statistical procedures detailed by the scale’s developer. The use of a single question from a multidimensional health survey to designate a cause-effect relationship to an AE is inappropriate.

## Evaluating AEs and SAEs

### Assessment of Intensity

The investigator will make an assessment of intensity for each AE and SAE reported during the study and will assign it to one of the following categories:

Mild: An event that is easily tolerated by the subject, causing minimal discomfort and not interfering with everyday activities.

Moderate: An event that is sufficiently discomforting to interfere with normal everyday activities.

Severe: An event that prevents normal everyday activities.

An AE that is assessed as severe will not be confused with an SAE. Severity is a category utilized for rating the intensity of an event; and both AEs and SAEs can be assessed as severe. An event is defined as ‘serious’ when it meets at least one of the pre-defined outcomes as described in the definition of an SAE.

### Assessment of Causality

The investigator is obligated to assess the relationship between investigational product and the occurrence of each AE/SAE. A "reasonable possibility" is meant to convey that there are facts/evidence or arguments to suggest a causal relationship, rather than a relationship cannot be ruled out. The investigator will use clinical judgment to determine the relationship. Alternative causes, such as natural history of the underlying diseases, concomitant therapy, other risk factors, and the temporal relationship of the event to the investigational product will be considered and investigated. The investigator will also consult the Investigator Brochure (IB) and/or Product Information, for marketed products, in the determination of his/her assessment.

There may be situations when an SAE has occurred and the investigator has minimal information to include in the initial report to GSK. However, **it is very important that the investigator always make an assessment of causality for every event prior to the initial transmission of the SAE data to GSK.** The investigator may change his/her opinion of causality in light of follow-up information, amending the SAE data collection tool accordingly. The causality assessment is one of the criteria used when determining regulatory reporting requirements.

## Follow-up of AEs and SAEs

After the initial AE/SAE report, the investigator is required to proactively follow each subject at subsequent visits/contacts. All AEs and SAEs will be followed until resolution, until the condition stabilizes, until the event is otherwise explained, or until the subject is lost to follow-up.

The investigator is obligated to perform or arrange for the conduct of supplemental measurements and/or evaluations as may be indicated or as requested by GSK to elucidate as fully as possible the nature and/or causality of the AE or SAE. The investigator is obligated to assist. This may include additional laboratory tests or investigations, histopathological examinations or consultation with other health care professionals. If a subject dies during participation in the study or during a recognized follow-up period, the investigator will provide GSK with a copy of any post-mortem findings, including histopathology. New or updated information will be recorded in the originally completed data collection tool. The investigator will submit any updated SAE data to GSK within the designated reporting time frames.

## Prompt Reporting of SAEs to GSK

Once the investigator determines that an event meets the protocol definition of an SAE, the SAE will be reported to GSK **within 24 hours**. Any follow-up information on a previously reported SAE will also be reported to GSK within 24 hours.

If the investigator does not have all information regarding an SAE, he/she will not wait to receive additional information before notifying GSK of the event and completing the appropriate data collection tool. The investigator will always provide an assessment of causality at the time of the initial report as described in Section 11.5.2, Assessment of Causality.

The primary mechanism for reporting SAEs to GSK will be the electronic data collection tool (e.g., PIMS).If the electronic system is unavailable for greater than 24 hours; the site will use the paper SAE data collection tool and fax it to the GSK Medical Monitor. Then the site will enter the serious adverse event data into the electronic system as soon as it becomes available.

After the study is completed at a given site, the electronic data collection tool (e.g., PIMS system) will be taken off-line to prevent the entry of new data or changes to existing data. If a site receives a report of a new SAE from a study participant or receives updated data on a previously reported SAE after the electronic data collection tool has been taken off-line, the site can report this information on a paper SAE form or to their GSK protocol contact by telephone.

GSK contacts for SAE receipt can be found at the beginning of this protocol on the Sponsor/Medical Monitor Contact Information page.

Facsimile transmission of the SAE data collection tool is the preferred method to transmit this information to the project contact for SAE receipt. In rare circumstances and in the absence of facsimile equipment, notification by telephone is acceptable, with a copy of the SAE data collection tool sent by overnight mail. Initial notification via the telephone does not replace the need for the investigator to complete and sign the SAE data collection tool within the designated reporting time frames.

GSK contacts for SAE receipt can be found at this beginning of this protocol on the Sponsor/Medical Monitor Contact Information page.

Facsimile transmission of the following PIMS listings for the corresponding subject is the preferred method to transmit SAE information to the GSK Medical Monitor or protocol contact:

- SAE listing
- Demographic listing
- Investigational product listing

In rare circumstances and in the absence of facsimile equipment, notification by telephone is acceptable, with a copy of all required information sent by overnight mail.

If the PIMS system is unavailable when the SAE occurs, the site will use the paper SAE form and fax that to the GSK medical Monitor or protocol contact. The site will enter the SAE data into PIMS as soon as the system becomes available.

## Regulatory Reporting Requirements For SAEs

Prompt notification of SAEs by the investigator to GSK is essential so that legal obligations and ethical responsibilities towards the safety of subjects are met.

GSK has a legal responsibility to notify both the local regulatory authority and other regulatory agencies about the safety of a product under clinical investigation. GSK will comply with country specific regulatory requirements relating to safety reporting to regulatory authorities, IRBs/IECs and investigators.

Investigator safety reports are prepared for suspected unexpected serious adverse reactions according to local regulatory requirements and GSK policy and are forwarded to investigators as necessary. An investigator who receives an investigator safety report describing an SAE(s) or other specific safety information (e.g., summary or listing of SAEs) from GSK will file it with the IB and will notify the IRB/IEC, if appropriate according to local requirements.

# MEDICAL DEVICES – INCIDENTS (INCLUDING MALFUNCTIONS)

GSK medical devices are being provided for use in this study. In order to fulfill regulatory reporting obligations worldwide the investigator is responsible for the detection and documentation of events meeting the definitions of incident or malfunction that occur during the study with such devices.

## Definitions of an Incident

The detection and documentation procedures described in this protocol apply to all GSK medical devices provided for use in the study (see Section 9.4 for the list of GSK medical devices).

**Incident** – Any malfunction or deterioration in the characteristics and/or performance of a device, as well as any inadequacy in the labeling or the instructions for use which, directly or indirectly, might lead to or might have lead to the death of a patient/user/other persons or to a serious deterioration in their state of health.

Not all INCIDENTS lead to death or serious deterioration in health. The non-occurrence of such a result might have been due to other fortunate circumstances or to the intervention of health care personnel.

It is sufficient that:

- an INCIDENT associated with a device happened and
- the INCIDENT was such that, if it occurred again, it might lead to death or serious deterioration in health.

A **serious deterioration in state of health** can include:

- life-threatening illness
- permanent impairment of body function or permanent damage to a body structure
- a condition necessitating medical or surgical intervention to prevent a) or b)
- fetal distress, fetal death or any congenital abnormality or birth defects

**Examples** of incidents

- a patient, user, care giver or professional is injured as a result of a medical device failure or its misuse
- a patient’s treatment is interrupted or compromised by a medical device failure
- misdiagnosis due to medical device failure leads to inappropriate treatment
- a patient’s health deteriorates due to medical device failure

## Time Period for Detecting Medical Device Incidents

Medical device incidents will be detected, documented and reported during all periods of the study in which the GSK medical devices are available for use. Additionally, malfunctions of the device that result in an incident will also be detected, documented and reported during all periods of the study in which the GSK medical devices are available for use.

If the investigator learns of any incident at any time after a subject has been discharged from the study, and such incident is reasonably related to a GSK medical device provided for the study, the investigator will promptly notify GSK.

## Documenting Medical Device Incidents

Any medical device incident occurring during the study will be documented in the subject’s medical records, in accordance with the investigator’s normal clinical practice, and on the appropriate form. **In addition, for incidents fulfilling the definition of an AE or an SAE, the appropriate AE/SAE data collection tool will be completed as previously described.**

The form will be completed as thoroughly as possible and signed by the investigator before transmittal to GSK. It is **very important that the investigator provides his/her assessment of causality to the medical device provided by GSK at the time of the initial report**, and describes any corrective or remedial actions taken to prevent recurrence of the incident. A remedial action is any action other than routine maintenance or servicing of a device where such action is necessary to prevent recurrence of an incident. This includes any amendment to the design to prevent recurrence.

## Follow-up of Medical Device Incidents

All medical device incidents involving an AE, will be followed until resolution of the event, until the condition stabilizes, until the condition is otherwise explained, or until the subject is lost to follow-up. This applies to all subjects, including those withdrawn prematurely. The investigator is responsible for ensuring that follow-up includes any supplemental investigations as may be indicated to elucidate as completely as practical the nature and/or causality of the incident.

New or updated information will be recorded on the originally completed form with all changes signed and dated by the investigator.

## Prompt Reporting of Medical Device Incidents to GSK

Medical device incidents will be reported to GSK within 24 hours once the investigator determines that the event meets the protocol definition of a medical device incident.

Facsimile transmission of the "Medical Device Incident Report Form" is the preferred method to transmit this information to the GSK project contact. The same individual will be the GSK project contact for receipt of medical device reports and SAEs. In the absence of facsimile equipment, notification by telephone is acceptable for incidents, with a copy of the "Medical Device Incident Report Form" sent by overnight mail.

## Regulatory Reporting Requirements for Medical Devices

The investigator will promptly report all incidents occurring with any GSK medical device provided for use in the study. GSK has a legal responsibility to notify appropriate regulatory bodies and other entities about certain safety information relating to medical devices being used in clinical studies. Prompt notification of incidents by the investigator to the appropriate project contact is essential in order to meet legal obligations and ethical responsibility towards the safety of subjects.

The investigator, or responsible person according to local requirements (e.g., the head of the medical institution in Japan), will comply with the applicable local regulatory requirements relating to the reporting of incidents to the IRB/IEC.

# Study CONDUCT CONSIDERATIONS

## Posting of Information on Clinicaltrials.gov

Study information from this protocol will be posted on clinicaltrials.gov before enrolment of subjects begins.

## Regulatory and Ethical Considerations, Including the Informed Consent Process

GSK will obtain favorable opinion/approval to conduct the study from the appropriate regulatory agency in accordance with any applicable country-specific regulatory requirements prior to a site initiating the study in that country.

The study will be conducted in accordance with all applicable regulatory requirements,

The study will also be conducted in accordance with "good clinical practice" (GCP), all applicable subject privacy requirements, and, the guiding principles of the 2008 Declaration of Helsinki. This includes, but is not limited to, the following:

- IRB/IEC review and favorable opinion/approval to conduct the study and of any subsequent relevant amended documents
- Written informed consent (and any amendments) to be obtained for each subject before participation in the study
- Investigator reporting requirements (e.g. reporting of AEs/SAEs/protocol deviations to IRB/IEC)

### Urgent Safety Measures

If an event occurs that is related to the conduct of the study or the development of the investigational product, and this new event is likely to affect the safety of subjects, the sponsor and the investigator will take appropriate urgent safety measures to protect subjects against any immediate hazard.

The sponsor will work with the investigator to ensure the IEC/IRB is notified.

## Quality Control (Study Monitoring)

In accordance with applicable regulations including GCP, and GSK procedures, GSK monitors will contact the site prior to the start of the study to review with the site staff the protocol, study requirements, and their responsibilities to satisfy regulatory, ethical, and GSK requirements. When reviewing data collection procedures, the discussion will also include identification, agreement and documentation of data items for which the PIMS record will serve as the source document.

GSK will monitor the study and site activity to verify that the:

- Data are authentic, accurate, and complete.
- Safety and rights of subjects are being protected.
- Study is conducted in accordance with the currently approved protocol and any other study agreements, GCP, and all applicable regulatory requirements.

The investigator and the head of the medical institution (where applicable) agrees to allow the monitor direct access to all relevant documents

## Quality Assurance

To ensure compliance with GCP and all applicable regulatory requirements, GSK may conduct a quality assurance audit. Regulatory agencies may also conduct a regulatory inspection of this study. Such audits/inspections can occur at any time during or after completion of the study. If an audit or inspection occurs, the investigator and institution agree to allow the auditor/inspector direct access to all relevant documents and to allocate his/her time and the time of his/her staff to the auditor/inspector to discuss findings and any relevant issues.

## Study and Site Closure

Upon completion or premature discontinuation of the study, the monitor will conduct site closure activities with the investigator or site staff, as appropriate, in accordance with applicable regulations including GCP, and GSK procedures.

In addition, GSK reserves the right to temporarily suspend or prematurely discontinue this study at any time for reasons including, but not limited to, safety or ethical issues or severe non-compliance. If GSK determines such action is needed, GSK will discuss this with the investigator or the head of the medical institution (where applicable), including the reasons for taking such action. When feasible, GSK will provide advance notification to the investigator or the head of the medical institution, where applicable, of the impending action prior to it taking effect.

If the study is suspended or prematurely discontinued for safety reasons, GSK will promptly inform investigators or the head of the medical institution (where applicable) and the regulatory authorities of the suspension or premature discontinuation of the study and the reason(s) for the action. If required by applicable regulations, the investigator or the head of the medical institution (where applicable) must inform the IRB/IEC promptly and provide the reason for the suspension or premature discontinuation.

## Records Retention

Following closure of the study, the investigator or the head of the medical institution (where applicable) must maintain all site study records, except for those required by local regulations to be maintained by someone else, in a safe and secure location. The records must be maintained to allow easy and timely retrieval, when needed (e.g., audit or inspection), and, whenever feasible, to allow any subsequent review of data in conjunction with assessment of the facility, supporting systems, and staff. Where permitted by local laws/regulations or institutional policy, some or all of these records can be maintained in a format other than hard copy (e.g., microfiche, scanned, electronic); however, caution needs to be exercised before such action is taken. The investigator must assure that all reproductions are legible and are a true and accurate copy of the original, and meet accessibility and retrieval standards, including re-generating a hard copy, if required. Furthermore, the investigator must ensure there is an acceptable back-up of these reproductions and that an acceptable quality control process exists for making these reproductions.

GSK will inform the investigator of the time period for retaining these records to comply with all applicable regulatory requirements. The minimum retention time will meet the strictest standard applicable to that site for the study, as dictated by any institutional requirements or local laws or regulations, or GSK standards/procedures; otherwise, the retention period will default to 15 years.

The investigator must notify GSK of any changes in the archival arrangements, including, but not limited to, archival at an off-site facility or transfer of ownership of the records in the event the investigator leaves the site.

## Provision of Study Results to Investigators, Posting to the Clinical Trials Register and Publication

Where required by applicable regulatory requirements, an investigator signatory will be identified for the approval of the clinical study report. The investigator will be provided reasonable access to statistical tables, figures, and relevant reports and will have the opportunity to review the complete study results at a GSK site or other mutually-agreeable location.

GSK will also provide the investigator with the full summary of the study results. The investigator is encouraged to share the summary results with the study subjects, as appropriate.

GSK will provide the investigator with the randomization codes for their site only after completion of the full statistical analysis.

The results summary will be posted to the Clinical Study Register at the time of the first regulatory approval or within 12 months of any decision to terminate development. In addition, a manuscript will be submitted to a peer reviewed journal for publication no later than 12 months after the first approval or any decision to terminate development. When manuscript publication in a peer reviewed journal is not feasible, further study information will be posted to the GSK Clinical Study Register to supplement the results summary.

The results summary for an approved GSK medicinal product will be posted to the Clinical Study Register no later than 12 months after the last subject’s last visit (LSLV) or sooner if required by legal agreement, local law or regulation. In addition, a manuscript will be submitted to a peer-reviewed journal for publication within 18 months of LSLV. When manuscript publication in a peer reviewed journal is not feasible, further study information is posted to the GSK Clinical Study Register to supplement the results summary.

A manuscript will be progressed for publication in the scientific literature if the results provide important scientific or medical knowledge.

## Data Management

GSK Data Management will identify and implement the most effective data acquisition and management strategy for each clinical trial protocol and deliver datasets which support the protocol objectives. Subject data will be entered into GSK defined CRFs and combined with data provided from other sources (e.g. diary data, laboratory data) in a validated data system. Subject initials will not be transmitted to GSK for inclusion in the datasets. Clinical data management will be performed in accordance with applicable GSK standards and data cleaning procedures with the objective of removing errors and inconsistencies in the data which would otherwise impact on the analysis and reporting objectives, or the credibility of the Clinical Study Report. Adverse events and concomitant medications terms will be coded using validated dictionaries. Original CRFs will be retained by GSK, while the investigator will retain a copy.

# References

Carter D, Howlett HC, Wiernsperger NF, Bailey CJ. Differential effects of metformin on bile salt absorption from the jejunum and iluem. *Diabetes, Obesity and Metabolism.* 2003;5(2):120-125.

Correia S, Carvalho C, Santos MS, Seica R, Oliveira CR, Moreira PI. Mechanisms of action of metformin in type 2 diabetes and associated complications: an overview. *Mini Review of Medical Chemistry.* 2008;Nov 8(13):1343-1354.

Glueck, CJ, Papanna, R, Wang, P, Goldenberg, N, Sieve-Smith, L Incidence and treatment of metabolic syndrome in newly referred women with confirmed polycystic ovarian syndrome. *Metabolism.* 2003 ;52: 908–15.

Kirpichnikov D, McFarlane SI, Sowers JR. Metformin: an update. *Annals of Internal Medicine.* 2002;Jul 2; 137(1):25-33.

Mannucci E, Tesi F, Bardini G, Ognibene A, Petracca MG, Ciani S, Pezzatini A, Brogi M, Dicembrini I, Cremasco F, Messeri G, Rotella CM. Effects of metformin on glucagon-like-peptide-1 levels in obese patients with and without Type 2 diabetes. *Diabetes Nutrition and Metabolism.* 2004;Dec;17(6):336-342.

Natali A, Ferrannini E. Effects of metformin and thiazolidinediones on suppression of hepatic glucose production and stimulation of glucose uptake in type 2 diabetes: a systematic review. *Diabetologia.* 2006;Mar:49(3):434-41. Epup Feb 14.

UK Prospective Diabetes Study (UKPDS) Group. Effect of intensive blood-glucose control with metformin on complications in overweight patients with type 2 diabetes. 1998; The Lancet, Volume 352, Issue 9131, Pages 854 – 865

Appendices

Appendix 1:Metformin EMC entry


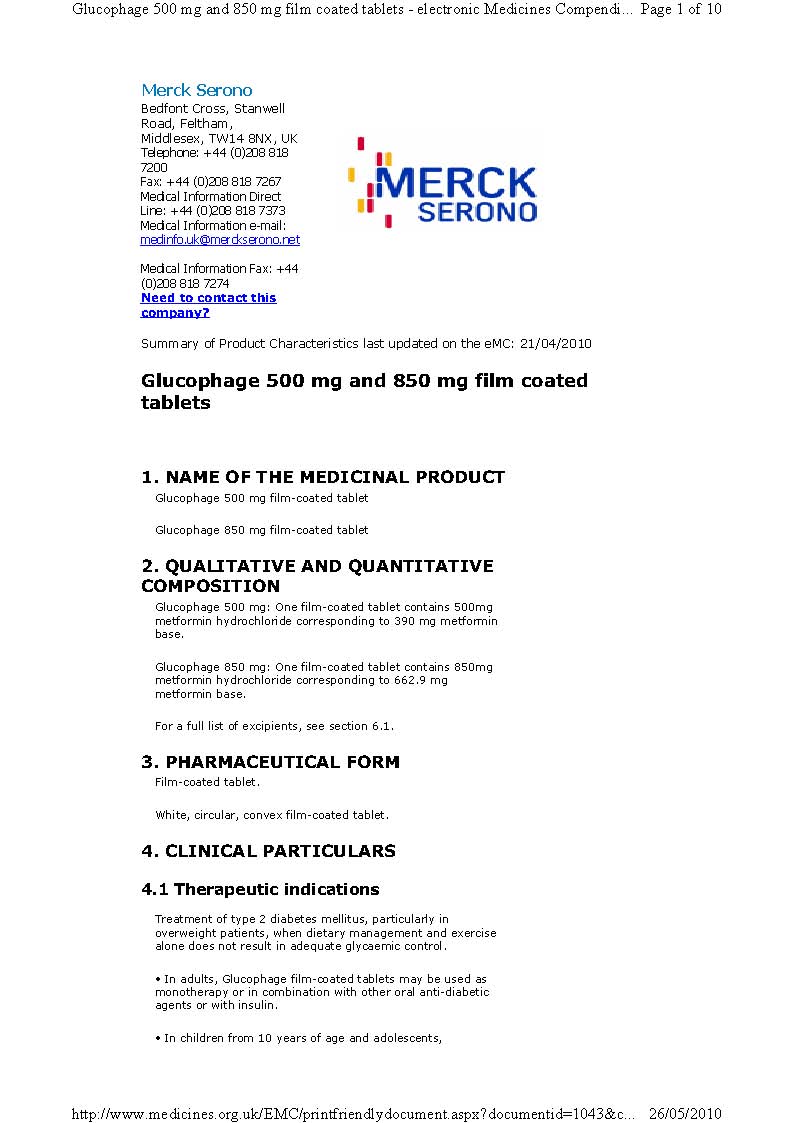


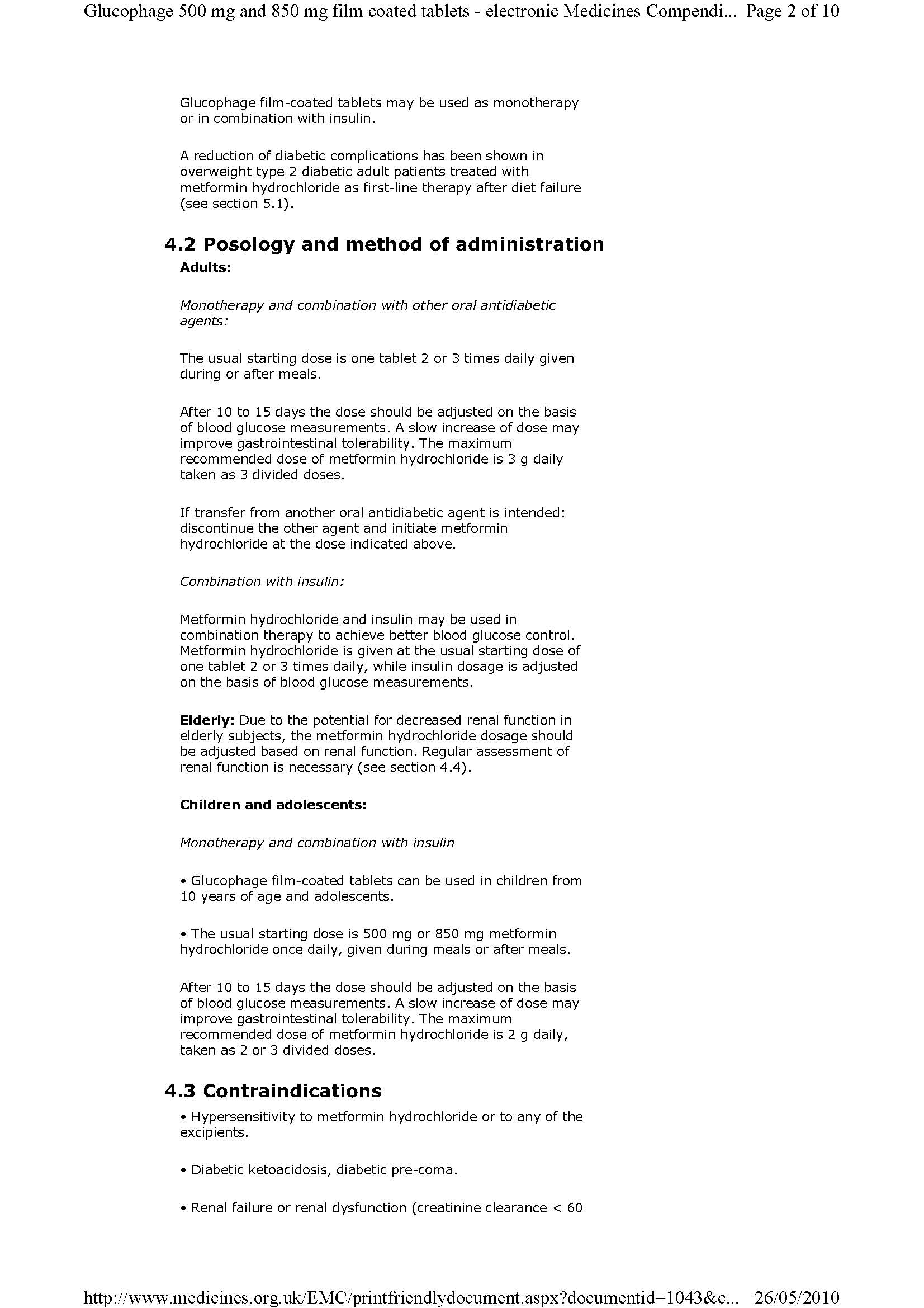


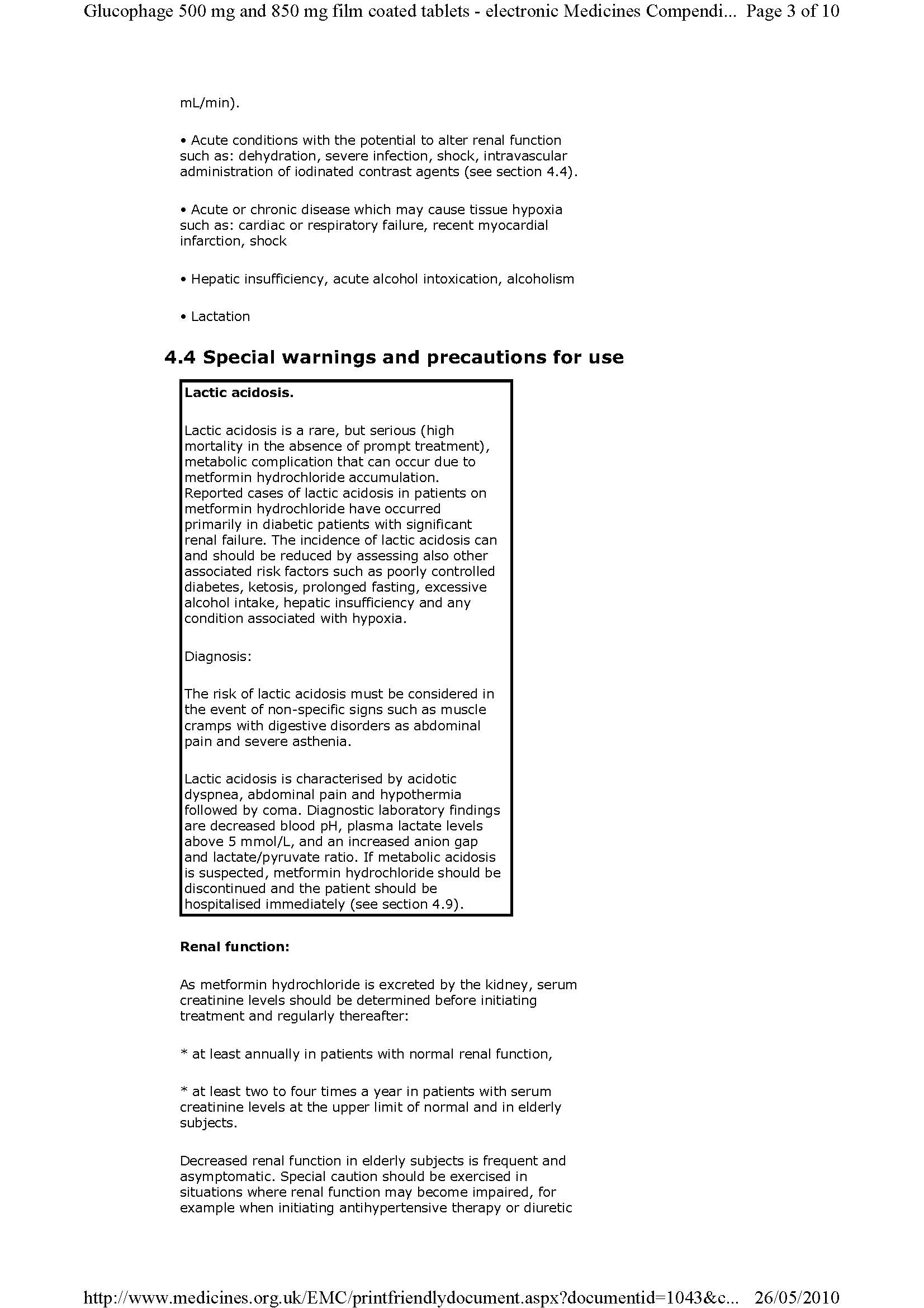


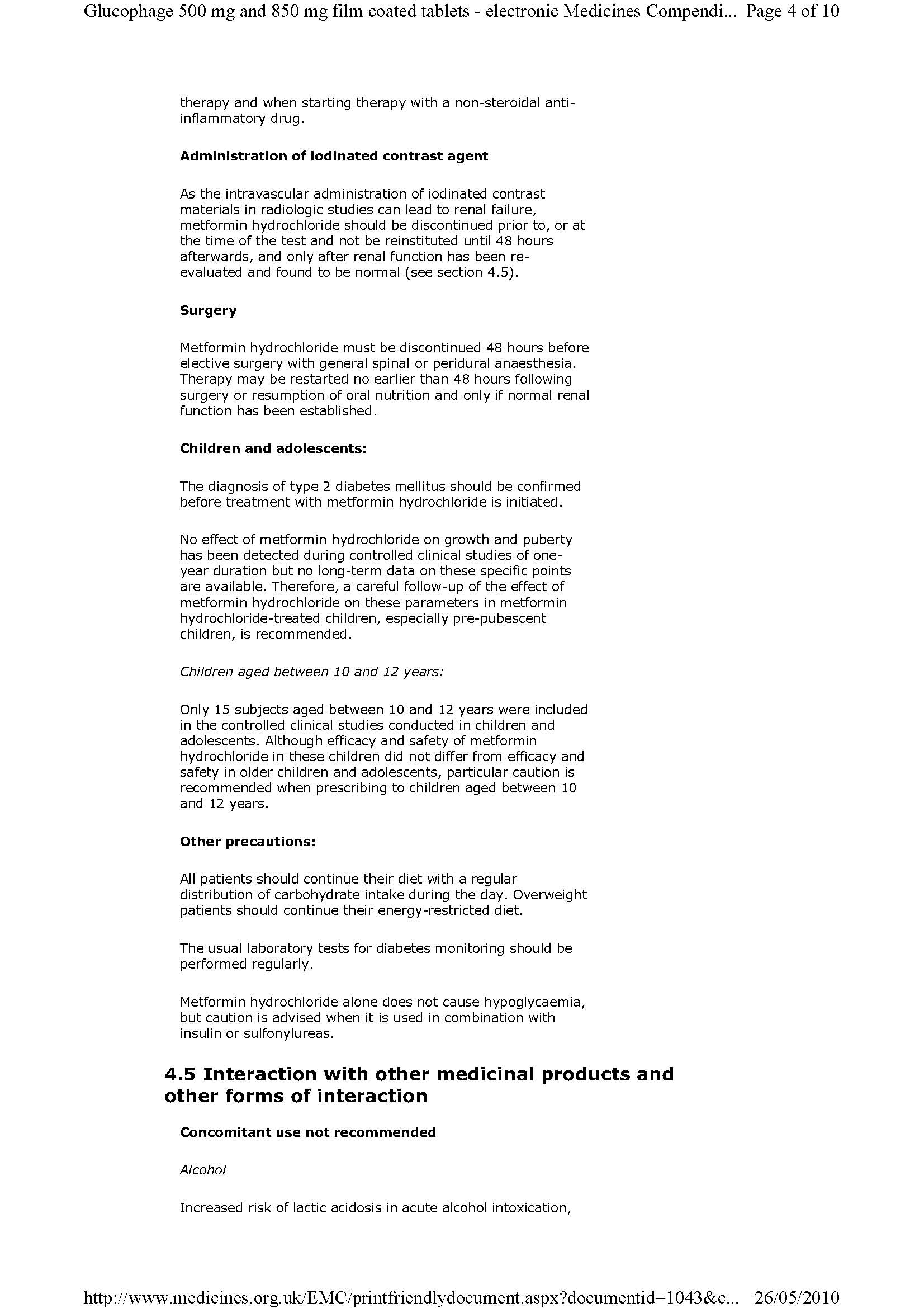


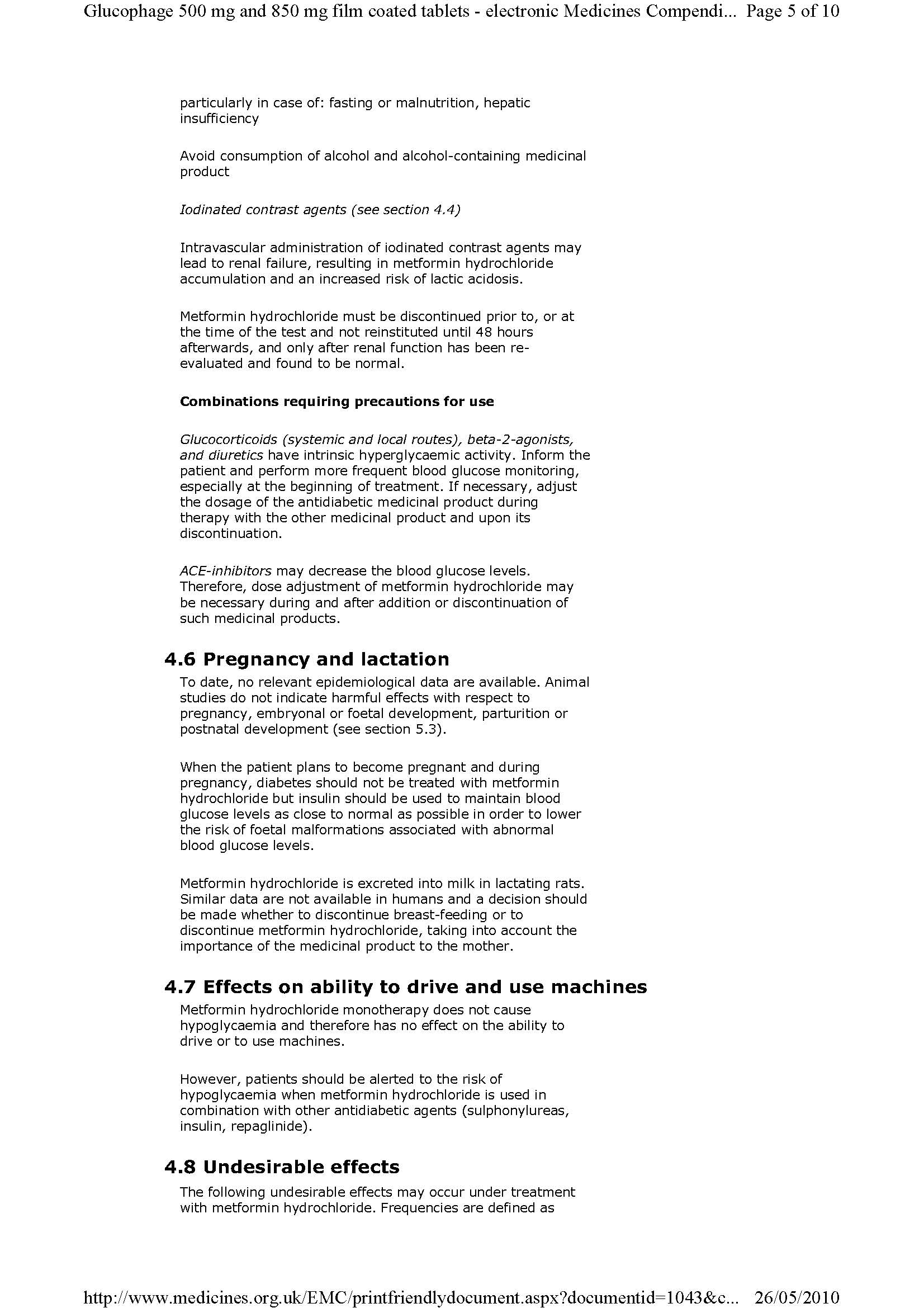


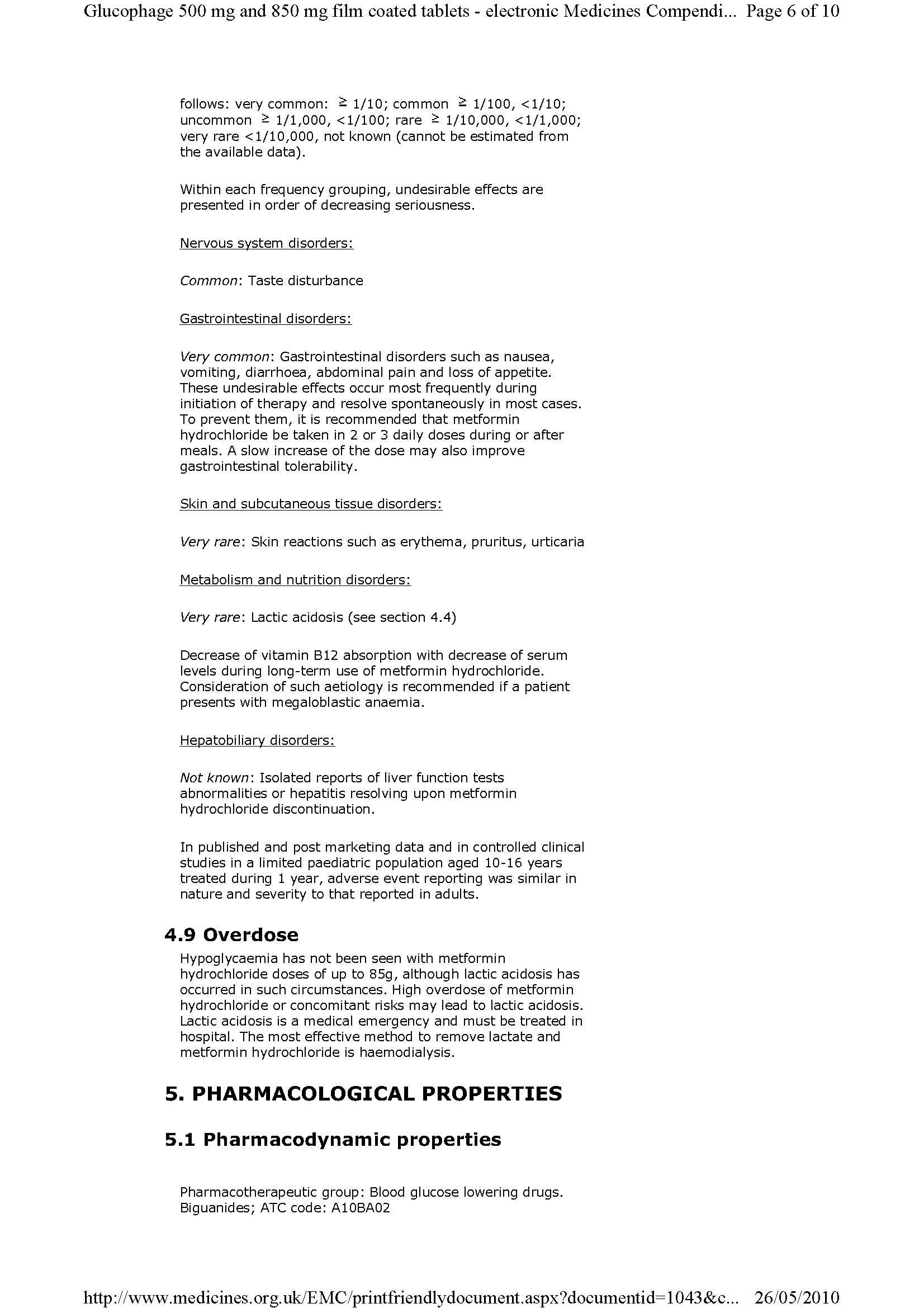


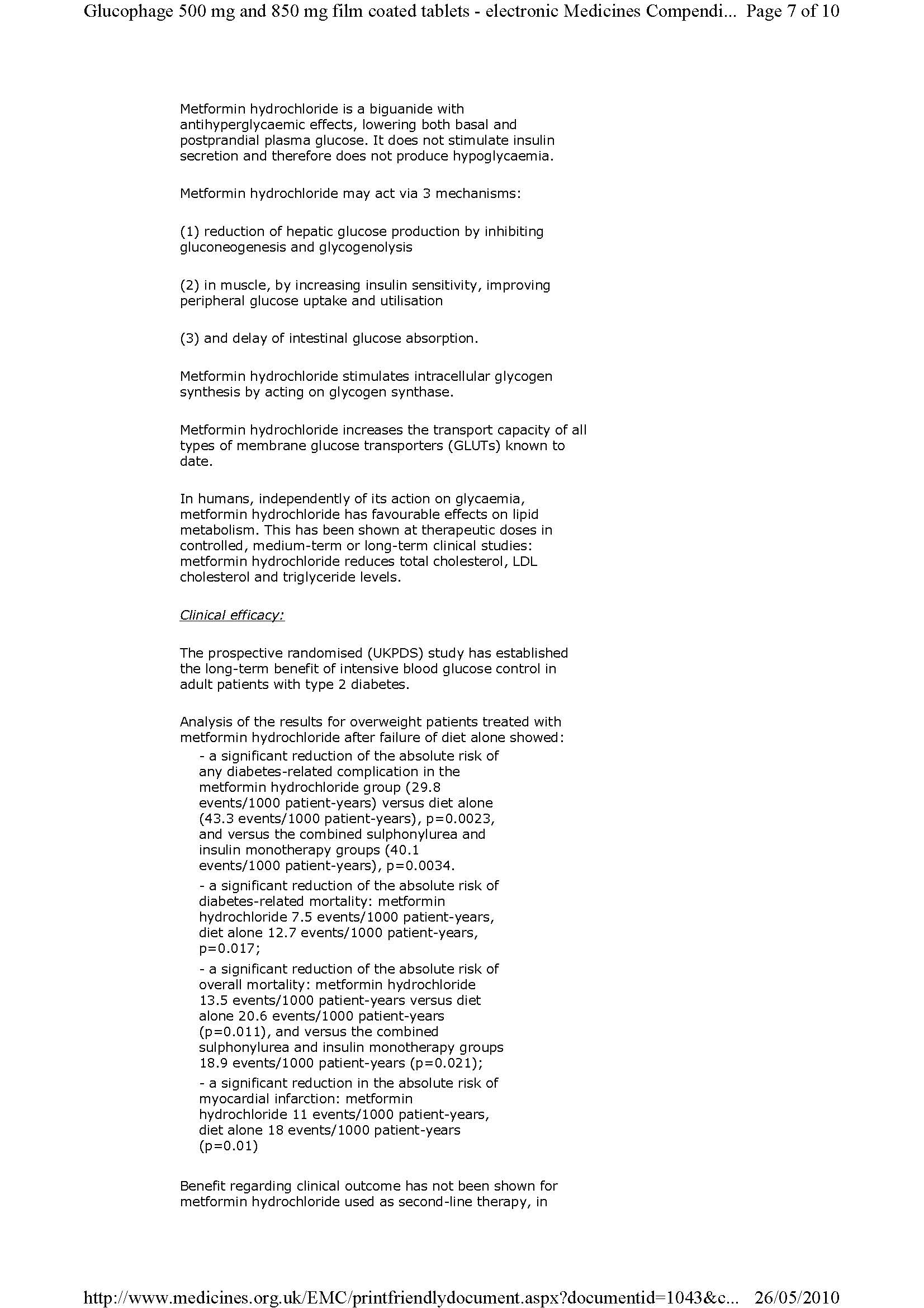


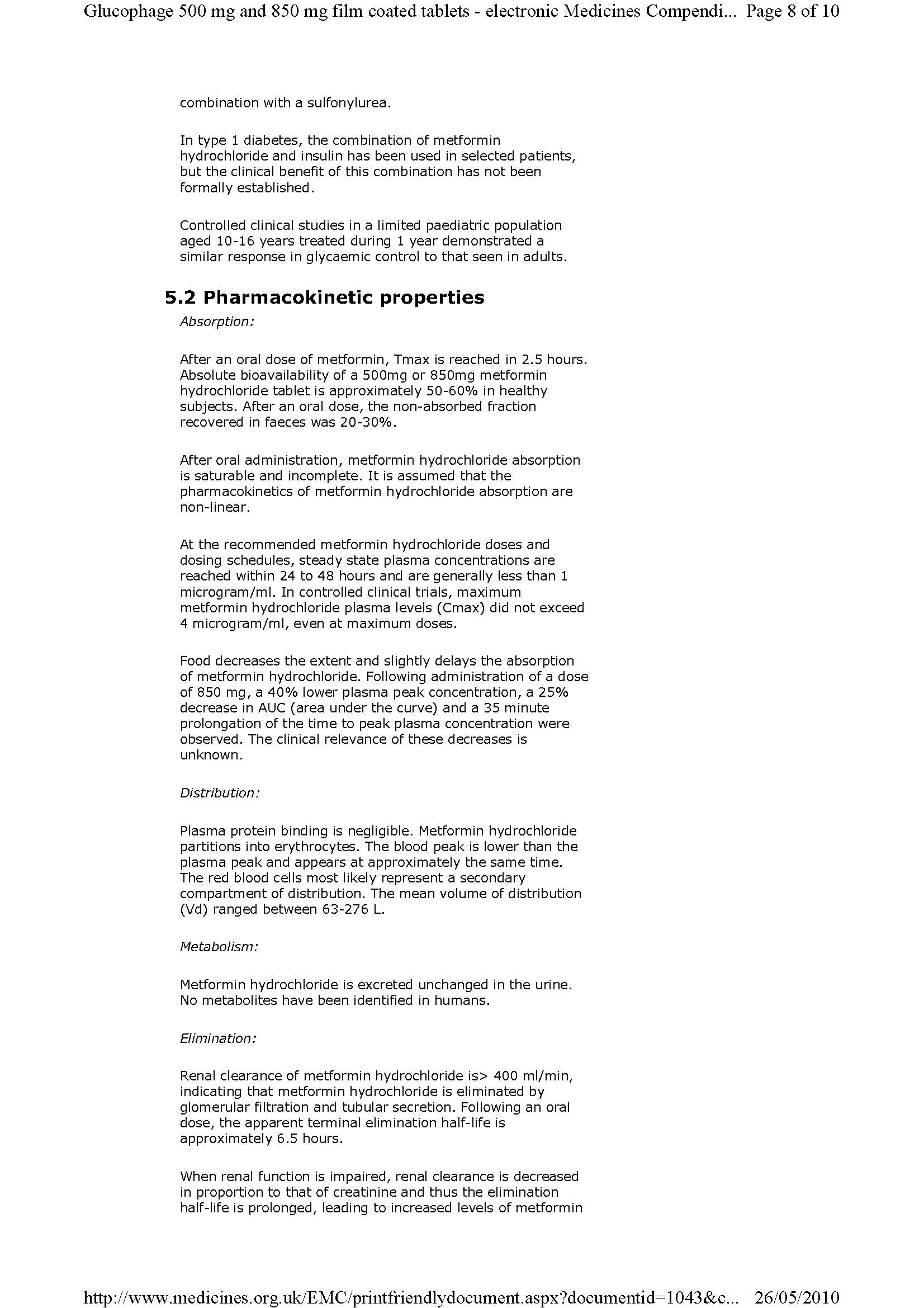


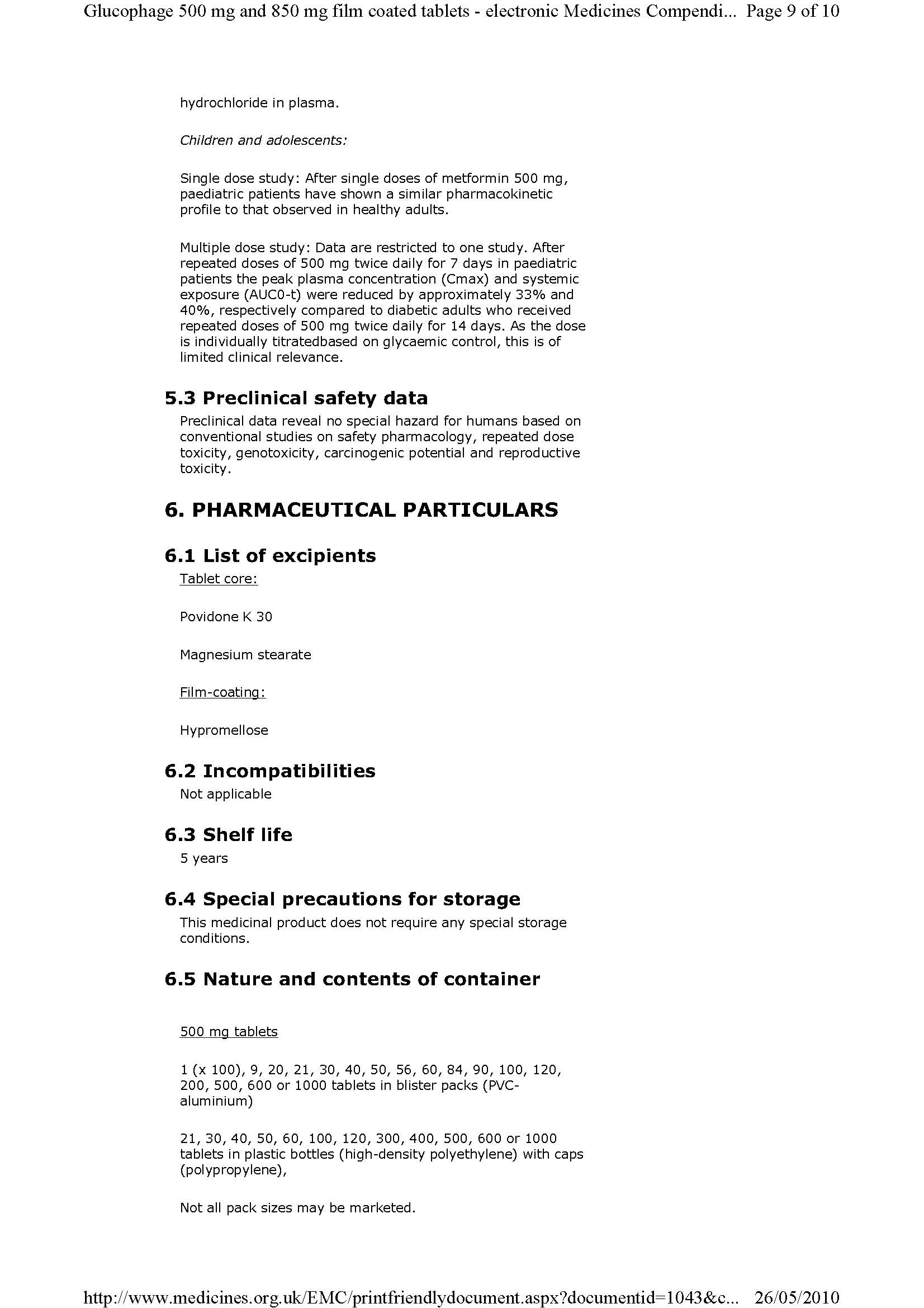


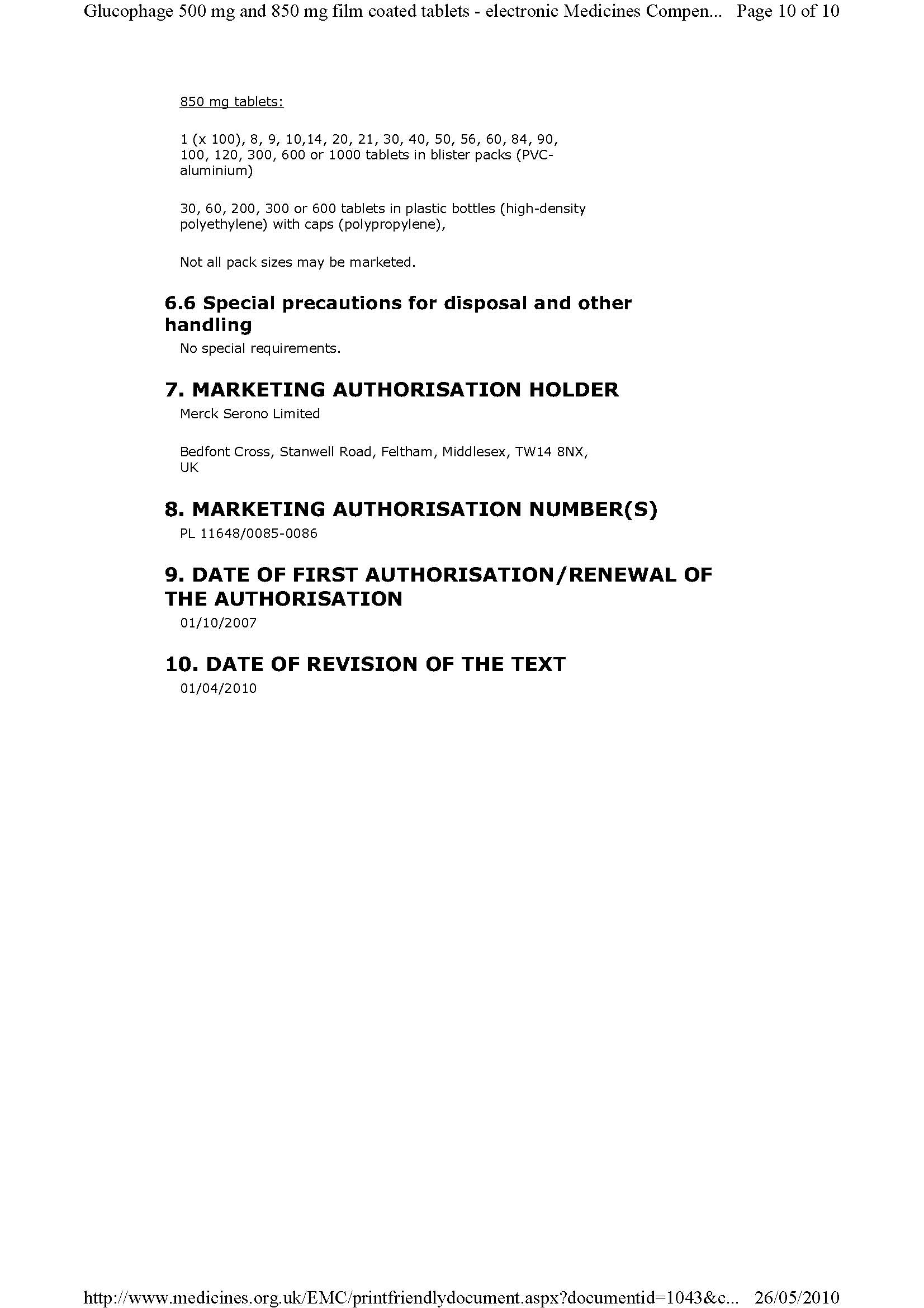

Supplement: Protocol S1 — Trial Protocol. (DOCX) [file pone.0100778.s002.docx]
